# Accessory Subunit Regulates Thiyl Radical Formation in Benzylsuccinate Synthase

**DOI:** 10.1021/acs.biochem.5c00492

**Published:** 2025-10-13

**Authors:** Shukurah Anas, Jian Liu, Anshika Vats, Rhea Gainadi, Siraj Sharif, Aiden Piriyatamwong, Mary Catherine Andorfer

**Affiliations:** Department of Chemistry, 3078 Michigan State University, East Lansing, Michigan 48824, United States

## Abstract

X-succinate synthases (XSSs) are a class of glycyl radical enzymes (GREs) that enable anaerobic hydrocarbon functionalization, granting anaerobes access to petroleum-derived substrates for metabolism. Owing to their ability to functionalize components of crude oil and catalyze selective olefin hydroalkylation, XSSs hold significant biotechnological promise. However, mechanistic understanding has been limited due to long-standing barriers to installing their essential glycyl radical in vitro, which have only recently been overcome. Unlike most GREs, XSSs contain accessory subunits that bind to the periphery of the catalytic subunit. The most well-studied XSS, benzylsuccinate synthase (BSS), includes two [4Fe–4S] cluster-binding accessory subunits, BSSγ and BSSβ. The full structure of BSSγ and the catalytic role of BSSβ have remained unclear. Here, we report the crystal structure of BSSγ with its [4Fe–4S] cluster intact, revealing a HiPP-like fold similar to that of BSSβ. Through biochemical and spectroscopic studies, we provide evidence that BSSβ promotes thiyl radical formation, even in the absence of a substrate. This finding contrasts with recent models, in which substrate binding is required to trigger thiyl radical formation. With this mechanistic insight, we optimized reaction conditions to achieve total turnover numbers of ∼17,000, representing an over 340-fold improvement compared to prior reports. We further show that in the absence of BSSβ, activated BSSαγ remains catalytically active for up to 11 days. Together, these results clarify the unique regulatory architecture of BSS and lay the groundwork for the use of XSSs in biocatalytic applications.

## Graphical abstract

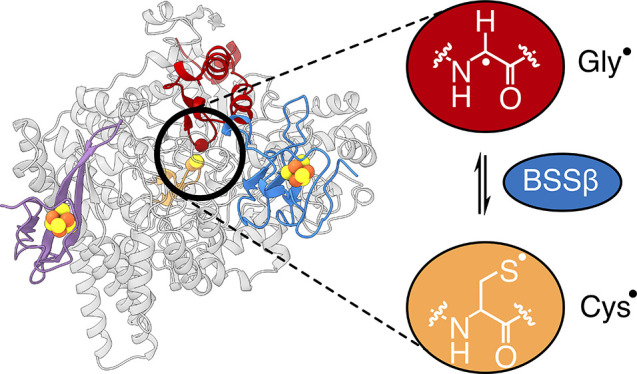

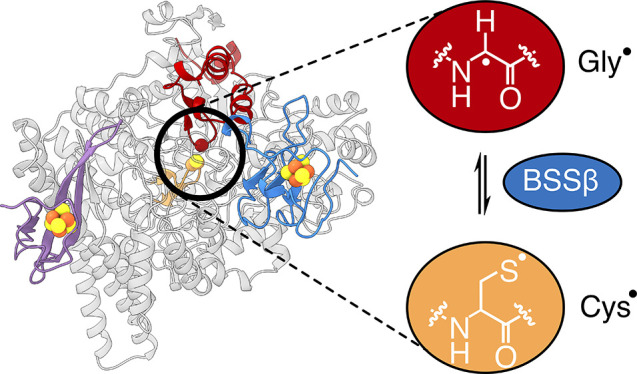

## Introduction

Glycyl radical enzymes (GREs) are a diverse superfamily that catalyze chemically challenging reactions in anaerobic microbes, playing key roles in central metabolism and environmental adaptation.^1,2^ Early characterized examples of GREs include pyruvate formate-lyase (PFL),^3^ benzylsuccinate synthase (BSS),^4–6^ and class III ribonucleotide reductase (aRNR).^7^ More recently, a growing number of GREs have been identified across diverse anaerobic environments, expanding their known reactivity.^1,8^ Their role in catalyzing a wide array of transformations, including those in the human microbiome, marine ecosystems, and oil reservoirs, makes GREs attractive targets for applications in human health, bioremediation, and biotechnology. Among them, BSS stands out for its ability to catalyze the stereoselective hydroalkylation of fumarate with toluene (Scheme 1),^9^ making it a compelling candidate for biocatalytic C–C bond formation. However, mechanistic analysis has been historically hindered by the inability to install its essential glycyl radical cofactor in vitro, which has only recently been overcome.^10,11^

**Scheme 1:**
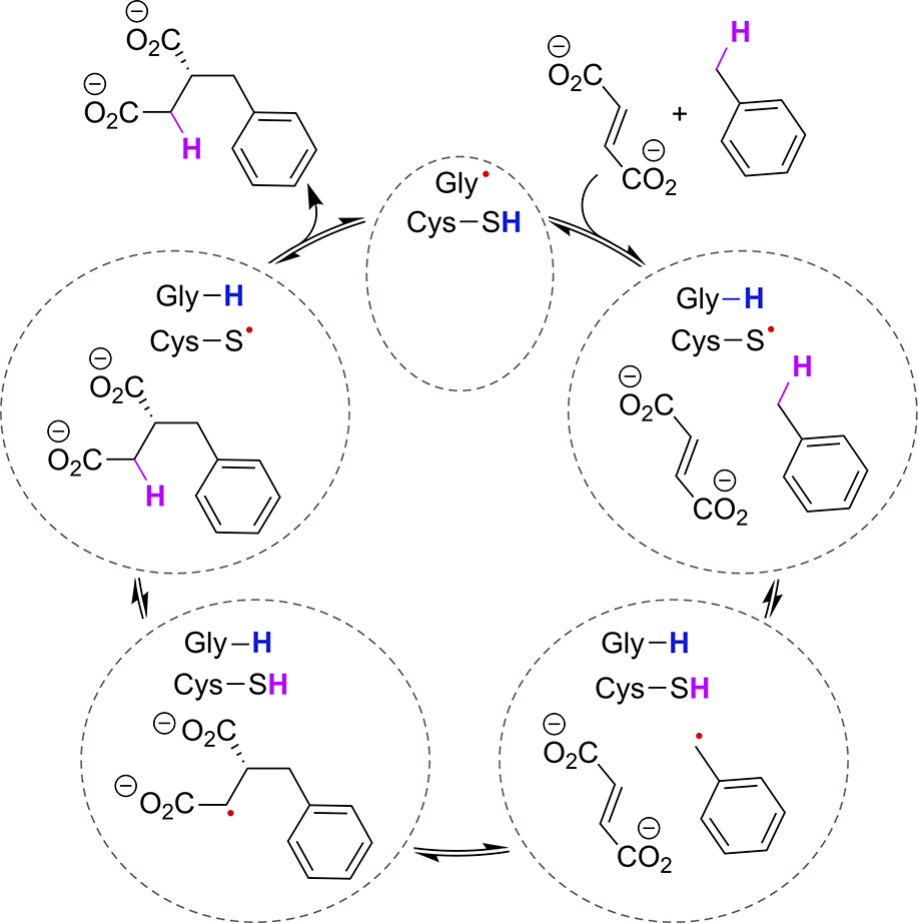
Catalytic Cycle of Benzylsuccinate Synthase (BSS)^a^

a BSS is a member of the glycyl radical enzyme (GRE) superfamily that catalyzes toluene addition to fumarate. A Gly· is post-translationally installed within BSS (Scheme 2). HAT can occur to transiently form a Cys·, which can abstract an H-atom from toluene. The resulting benzylic radical can add to fumarate to form a benzylsuccinate radical, which reabstracts the H-atom from Cys. The *R*-benzylsuccinate product is released from the active site, and the Cys· reabstracts an H-atom from Gly, where the radical is stored for the next round of turnover. Gray dashed circles represent the active site of BSS.

Canonical GREs contain conserved glycine and cysteine residues that are essential for catalysis,^1,2,12^ but recent work has expanded the superfamily to include enzymes in which the essential Gly is replaced by other residues (e.g., Ser, Ala, and Thr).^13^ Within canonical GREs, the glycyl radical (Gly·) is generated on the conserved Gly by an activating enzyme (AE) and subsequently transferred to Cys, forming a thiyl radical (Cys·) that initiates substrate activation (Scheme 2). These residues are housed within a 10-stranded β/α-barrel: the Gly resides in a loop located in the glycyl radical domain (GRD) near the periphery of the barrel, while the Cys is positioned in a loop near the active site within the barrel. The GRD has been shown to be flexible in GREs, a feature critical for glycyl radical formation, as large conformational changes are required to position the essential Gly residue within the active site of the AE.^14–17^ Detailed mechanistic characterization has been described for many of these enzymes, but due to difficulties in handling, key questions about this superfamily remain. One such question centers on how glycyl radical installation and subsequent hydrogen atom transfer (HAT) to form Cys· are regulated.^14,18^ To date, no structural data exist for a GRE:AE complex, and our understanding of the intermediate steps between glycyl radical formation and the emergence of a catalytically competent state capable of generating thiyl and substrate radicals remains limited.

**Scheme 2:**
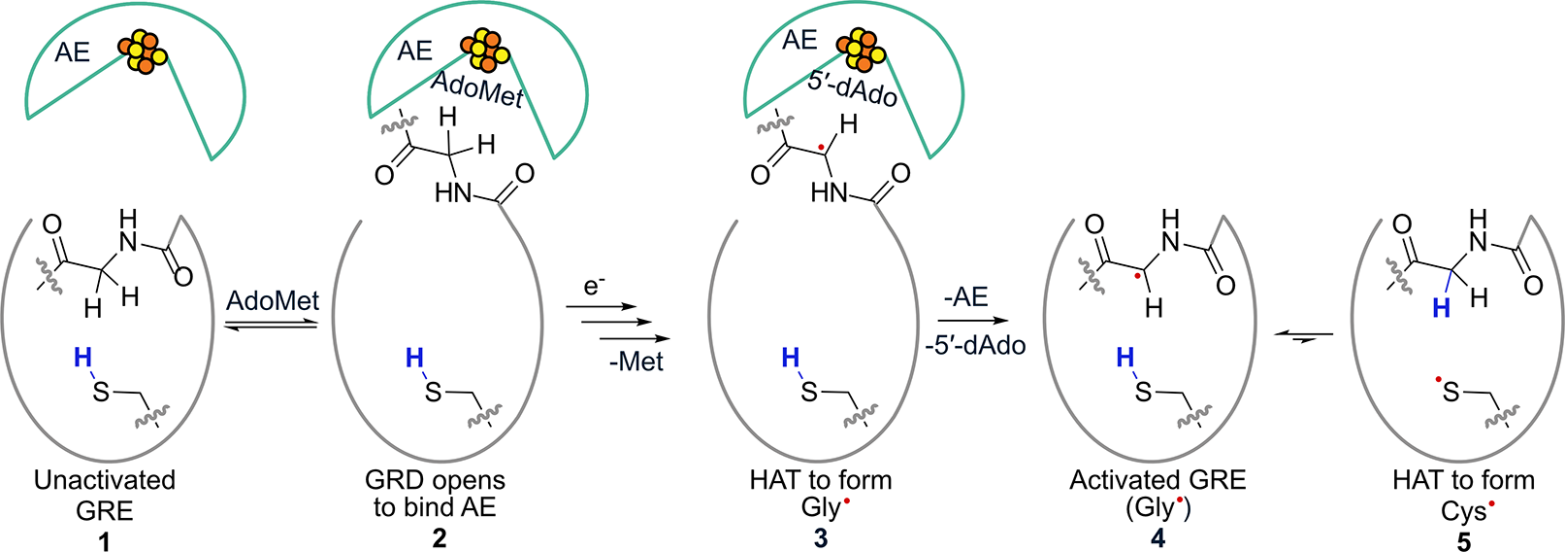
Overview of Glycyl Radical Installation within GREs^a^

a GREs can adopt closed and open conformations (**1** and **2**, respectively). In the open conformation, the glycyl radical domain (GRD) moves out of the active site to interact with the AdoMet-dependent activating enzyme (AE). The reduced [4Fe–4S]^+^ cluster in the AE active site can reductively cleave AdoMet to form a 5′-dAdo radical. This radical can abstract an H-atom from the essential Gly of the GRE, thus generating the glycyl radical (**3**). For catalysis to occur, the GRD must adopt the closed conformation (**4**) that brings the Gly and Cys residues in close enough proximity for HAT to occur, thus forming the transient thiyl radical (**5**).

A system that allows direct comparison between the glycyl radical (Gly·) and thiyl radical (Cys·) states would provide a useful platform to investigate radical persistence in vitro and clarify how radical transfer to cysteine influences the overall catalyst lifetime. Although thiyl radicals have not been directly observed in GREs, the glycyl radical within these enzymes has been extensively characterized by electron paramagnetic resonance (EPR) spectroscopy.^7,12,19–22^ Typically, EPR spectra show a doublet signal arising from the Gly·, suggesting that the radical equilibrium lies heavily toward the glycyl radical state. This finding is consistent with known reduction potentials and bond dissociation energies of glycine and cysteine.^23,24^ Though the long-lived nature of the Gly· is a hallmark of GREs, its lifetime in vitro is rarely measured and can vary significantly depending on reaction conditions.^17,25–27^ Many notable examples have been documented for pyruvate formate-lyase (PFL). The half-life of the Gly· in PFL is estimated to be over 24 h, though this lifetime can be perturbed by changes to reaction conditions such as [PFL-AE] and [reductant].^17,25,28,29^ In some cases, direct quenching of the glycyl radical has been observed,^30^ whereas in other cases, the more reactive Cys· is quenched.^31^ A clearer understanding of how these radical states interconvert and how their activated catalyst lifetimes are affected remains a critical gap in our understanding of GRE function.

Current data indicate that most GREs, including PFL, are homodimers composed of a single polypeptide sequence;^1,32^ however, benzylsuccinate synthase (BSS, Scheme 1)^15,33–35^ and 4-hydroxyphenylacetate decarboxylase (HPAD)^36,37^ also contain small accessory subunits (7–10 kDa) that bind [4Fe–4S] clusters (Figure S1). The reason for this variation in subunit architecture across GREs remains unclear, and it is possible that additional accessory subunits in other GREs have yet to be identified or annotated. BSS and HPAD both bind a small, accessory γ subunit, neither of which has been implicated in catalysis but instead is thought to serve a structural role. The γ subunit of BSS (BSSγ) has not been fully visualized but is proposed to adopt a HiPP-like fold.^15^ The large catalytic subunit (BSSα) cannot be obtained as soluble protein without BSSγ, and as such, no additional function has been proposed beyond solubility.^15,33^

BSS also binds a small β subunit (BSSβ), which is currently the only accessory subunit implicated in catalysis (Figure 1).^10^ Structural characterization of BSS revealed that BSSβ resides on the surface of the α subunit, >30 Å from the catalytic Cys residue.^15,34^ Based on comparative structures of BSS with and without the β subunit,^15^ several potential roles for BSSβ were proposed: (1) regulating conformational changes required for radical installation by the activating enzyme (AE), (2) promoting Cys· formation, and (3) gating substrate (toluene) access to the active site. We have recently observed that significant amounts of glycyl radical are only installed in BSSα when BSSβ is omitted from activation reactions, but that BSSβ is necessary for more than trace levels of hydroalkylation to occur.^10^ These results are consistent with the roles of BSSβ proposed above but do not rule out alternate explanations. Others have suggested an alternate role for BSSβ, where the [4Fe–4S] cluster within BSSβ may serve a protective function by acting as the terminal reductant in glycyl radical reduction to glycine in the absence of substrates.^38^ More recently, computational modeling suggested that Cys· formation in BSS, and potentially in all GREs, is triggered by substrate binding, rather than by the β subunit.^18^

**Figure 1:**
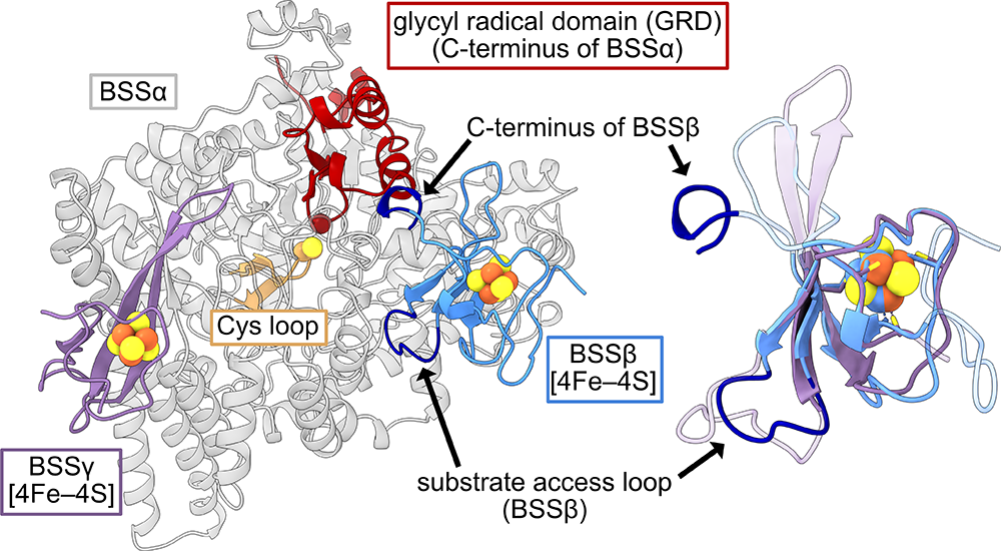
Structure of BSSαβγ, including previously unresolved regions of BSSγ and its [4Fe–4S] cluster, and overlay of BSSγ and BSSβ. This structure of BSSαβγ closely resembles those previously published but includes resolved density for the BSSγ subunit (purple). BSSγ has no known catalytic function but is known to promote solubility of the BSSα subunit (gray and red). Structurally, BSSγ resembles BSSβ (blue), with both adopting HiPIP-like folds. Despite this similarity, BSSβ has been proposed to play multiple roles in catalysis, including gating substrate (toluene) access and regulating conformational changes in the glycyl radical domain (GRD, red).

To better understand the roles of the accessory subunits in BSS, we solved the structure of the full complex, visualizing the complete BSSγ subunit and its [4Fe–4S] cluster for the first time. Building on the recent ability to activate BSS in vitro, we then turned to EPR spectroscopy and deuterium exchange studies to examine the formation of Cys· in the presence and absence of BSSβ. These studies revealed that BSSβ is required for the formation of significant quantities of Cys· within the catalytic subunit. This stands in stark contrast to PFL, where rapid H/D exchange indicative of Cys· formation is readily observed.^17,19,31,39^ By leveraging this ability to control the timing of Cys· formation within BSS, we show that hydroalkylation activity is retained for over 11 days in vitro when BSSβ is omitted during activation, consistent with a long-lived glycyl radical under these conditions. In contrast, inclusion of BSSβ substantially shortens the radical lifetime. These results suggest differential stability of radical-associated states, consistent with a core feature of GRE catalysis: a long-lived Gly· that serves as a radical reservoir and a short-lived, highly reactive Cys· that initiates catalysis.

With this mechanistic insight, we optimized hydroalkylation conditions by tuning BSSβ concentration and its timing of addition. These changes dramatically extended catalyst lifetime and lowered catalyst loadings, enabling a >340-fold improvement in total turnover number (TTN) compared to previous reports.^10^ Together, these results provide insight into radical regulation within BSS and lay the groundwork for engineering XSS enzymes for selective biocatalytic transformations.

## Methods

### Protein Expression and Purification

Genes and expression plasmids used in this study are detailed in the Supporting Information. Expression and purification of proteins are described previously.^10,11^ Briefly, plasmids were used to transform *E. coli* BL21 (DE3) competent cells (T7 Express cells, New England BioLabs). Glycerol stocks were prepared from single colonies. Starter cultures were grown overnight at 37 °C and 220 rpm in LB media with the appropriate antibiotics. Expression cultures were inoculated 1:100 into LB (for BSSαγ and BSSβ) or TB (for IbsAE), which was supplemented with antibiotics. All expression cultures, except those inoculated with the BSSβ-ΔFeS variant, were also supplemented with Fe­(II) ammonium sulfate and l-cysteine. Expression cultures were grown at 37 °C, 220 rpm to an OD_600_ of 0.8–1.0, induced with 1 mM IPTG, and incubated overnight at 22 °C, 100 rpm. Cells were harvested by centrifugation, flash-frozen in liquid nitrogen, and stored at −80 °C. All subsequent lysis and purification steps were carried out anaerobically at 10 °C in an MBraun glovebox. Frozen cell pellets were thawed and resuspended in sparged lysis buffer (50 mM HEPES, pH 8.0, 300 mM NaCl, 10% glycerol) containing lysozyme and DNase I and then lysed by sonication. Lysates were clarified by centrifugation and applied to gravity-packed columns containing Ni Sepharose Excel resin. Proteins were eluted (50 mM HEPES, pH 8.0, 300 mM NaCl, 300 mM imidazole), buffer-exchanged into activation/desalting buffer (50 mM HEPES, pH 8.0, 300 mM NaCl), and concentrated to ∼200–500 μM. Proteins were aliquoted, flash-frozen in liquid nitrogen, and stored at −80 °C. Iron content was quantified using the ferene assay.^40^

### Crystallography

BSSαβγ crystals were obtained using the sitting-drop vapor diffusion method under conditions similar to previous reports.^34^ Purified BSSαγ and purified BSSβ (50 mM HEPES pH 8.0, 300 mM NaCl) were mixed in a 1:2 molar ratio to a final concentration of 20 mg/mL. Crystallization screens were set in 24 well plates (Hampton research cat. no. HR3-160) inside an anaerobic Coy chamber filled with 97.5% N_2_ and 2.5% H_2_, with the O_2_ level measuring ∼5 ppm. Protein mixture (2.5 μL) and well solution (2.5 μL) were mixed and screened against 1 mL of well solutions, containing 100 mM Tris (pH 8.5), 21–31% (w/v) PEG3350, 60–90 mM KCl, and 5 mM fumarate. After 2–3 weeks, light-brown BSSαβγ crystals appeared. The crystal used for structural analysis was obtained from a condition containing 29% (w/v) PEG 3350, 60 mM KCl, 100 mM Tris (pH 8.5), and 5 mM fumarate. After incubation at room temperature for 4 weeks, BSSαβγ crystals were harvested and flash-frozen under anaerobic conditions inside the Coy chamber. Prior to freezing, crystals were soaked for 30 s in a cryoprotectant solution containing 100 mM Tris (pH 8.5), 50 mM HEPES (pH 8.0), 29% (w/v) PEG 3350, 35% (w/v) PEG 400, 300 mM NaCl, and 5 mM fumarate.

X-ray diffraction data were collected at NSLS II NYX (19-ID) at Brookhaven National Lab by LS-CAT (APS) members. 1800 frames (0.2°/frame, total 360°) were collected from a single crystal with an Eiger2 9 M XE detector. The data set was processed and merged using XDS^41^ and the CCP4 suite.^42^ The BSSαβγ crystal structure was solved with molecular replacement using 5BWE.^34^ Structure refinement was performed in Phenix^43^ and followed by manual model rebuilding and modification in Coot^44^ against the 2Fo-Fc and Fo-Fc maps. Data collection and refinement statistics for the structure reported here are listed in Table S1. All figures were prepared with PyMol (v2.5.2, Schrödinger, LLC) or ChimeraX.^45^

### Activations (Glycyl Radical Installation)

Detailed description of activation reactions is described previously.^11^ Activation reactions were conducted in an MBraun anaerobic chamber with an activated carbon filter to remove atmospheric toluene. Reactions contained DTT (1 mM final concentration), 5-deazariboflavin (50 μM final concentration), IbsAE (50 μM final concentration), BSSαγ (50 μM final concentration), and AdoMet (1.5 mM final concentration, Sigma Aldrich CAS 86867-01-8) diluted with activation buffer (50 mM HEPES pH 8.0, 300 mM NaCl) to the desired final volume (between 25 and 1000 μL). The activation reaction was gently mixed and incubated in a metal heat block at 25 °C under a standard fluorescent front-mounted lamp provided with the MBraun for 3–24 h. Activation reactions were used in hydroalkylation reactions or EPR assays (see below).

### Hydroalkylations

Following the installation of glycyl radical in BSSαγ, hydroalkylation reactions were initiated by adding DTT (1 mM final concentration), fumarate (2–10 mM final concentration), toluene (0.5–2% v/v), BSSβ (10–80 μM final concentration), and activated BSSαγ (0.25–2 μM final concentration, added as activation reaction) diluted to the final reaction volume with activation buffer (50 mM HEPES pH 8.0, 300 mM NaCl). Hydroalkylation reactions were removed from the anaerobic chamber and quenched with two volumes of methanol. Internal standard was added (one volume of 2 mM 3-chlorobenzoic acid). The precipitated protein was pelleted via centrifugation, and the supernatant was filtered through a 0.22 μm filter for LC/MS analysis. Samples were diluted as needed for MS analysis. Details of MS analysis are found in the Supporting Information.

### EPR Assays

EPR spectra of the glycyl radical were collected at 120 K on a Bruker EMX-Plus spectrometer. Spectra were recorded at 9.31 GHz with a modulation amplitude of 3 G, a microwave power of 1.26 μW, and a modulation frequency of 100 kHz. Spectra were collected with a center field of 3320 G, a sweep time of 60 s, and a sweep width of 200 G. Each spectrum shown is an average of 10 scans. Samples for EPR were prepared and flash-frozen in the MBraun chamber under anaerobic conditions. Undiluted activation reactions were used for glycyl radical quantification. For H/D exchange experiments, activation reactions were diluted to varying extents in buffer made using either H_2_O or D_2_O, after which small molecules and/or purified subunits were added. Detailed conditions for each experiment are provided in the figure legends.

## Results

### BSSγ Adopts a HiPIP-like Fold

As noted above, the structure of BSS­(αβγ)_2_ has been previously solved; however, in all reported structures, the N-terminus, C-terminus, and [4Fe–4S] cluster of the γ subunit are unresolved.^15,34^ We modified the protein purification protocol and used previously established crystallization conditions to obtain a structure of the full BSS­(αβγ)_2_ complex to 2.9 Å resolution (Figure 1, Table S1). Notably, our purification approach allowed us to resolve the majority of the BSSγ subunit (residues 5–59 out of 60), and we observed clear density for the [4Fe–4S] cluster known to bind this subunit (Figure S2A,B).^15,33^ Based on prior modeling and cyclic voltammetry experiments, BSSγ was proposed to adopt a high potential iron-sulfur protein (HiPIP) fold;^15^ our structure confirms this proposed architecture (Figure 1, overlay of β and γ). We also observe clear density for BSSβ (residues 13–81 out of 81) and its associated [4Fe–4S] cluster (Figure S2C). It has previously been proposed that a loop within BSSβ controls hydrocarbon access to the active site while the C-terminus controls conformational changes of the GRD that could be associated with formation of the thiyl radical and/or activation by AE (Figure 1).^10,15,34^ The catalytic BSSα subunit adopts the characteristic 10-stranded β/α-barrel and exhibits high structural similarity to previously reported structures (R.M.S.D. = 0.183, residues 9–865 out of 865). In the active site, both toluene and fumarate were modeled and align well with prior structures (Figure S2D); however, only fumarate was intentionally included in the crystallization conditions. The remaining electron density in the toluene-binding pocket was too large to model as water, and toluene provided the best fit. Although toluene was not added to the crystallization conditions, it is present in the atmosphere of our anaerobic chamber and binds the BSSαβγ complex with high affinity.^34,46^ Consistent with its strong binding, we observe benzylsuccinate formation in control reactions lacking added toluene, provided that all other components are present. These findings prompted us to equip our anaerobic chamber with an activated-carbon solvent filter, which eliminated contamination in subsequent experiments.

### Effect of BSSβ on Catalyst Lifetime

We had previously observed that activating BSS in the presence of BSSβ significantly reduces glycyl radical formation.^10^ We therefore developed a two-step protocol in which BSSαγ is first activated in the absence of BSSβ, and hydroalkylation reactions are subsequently conducted with purified BSSβ added.^10,11^ It is important to note that no purification is conducted between activation and hydroalkylation, and thus, AE is always present in hydroalkylations. Using these assays, we reported high assay yields for toluene addition to fumarate using BSSαβγ; however, these high yields required high catalyst loadings (2 mol % BSSαγ). Time-course experiments revealed that short catalyst lifetime, rather than slow turnover rates, was responsible for high loading requirements (Figure S3). Upon optimizing hydroalkylation conditions, we identified two key factors that influence catalyst lifetime: the timing of BSSβ addition and its concentration. At low catalyst loadings (0.05 mol % BSSαγ), the effect of BSSβ addition becomes evident; when BSSβ is added to activated BSSαγ prior to substrate addition, only trace levels of product are observed (Figure 2A, Table S2). In contrast, when BSSβ is added to activated BSSαγ in the presence of substrates, robust product formation occurs. Catalyst performance is also sensitive to the concentration of BSSβ. Higher [BSSβ] results in greater variability in product yield, even when BSSβ is added to hydroalkylations after substrates (Figure S4). The most consistent and prolonged activity is observed when BSSβ is added after substrates and used at lower concentrations. Under these conditions, hydroalkylation typically proceeds for up to 10 h (Figure 2B, Table S3). Lower catalyst loadings can be used to achieve high yields (0.01 mol% BSSαγ), and significantly higher total turnover numbers (TTN) are observed (TTN ∼ 17,000, an over 340-fold improvement).

**Figure 2:**
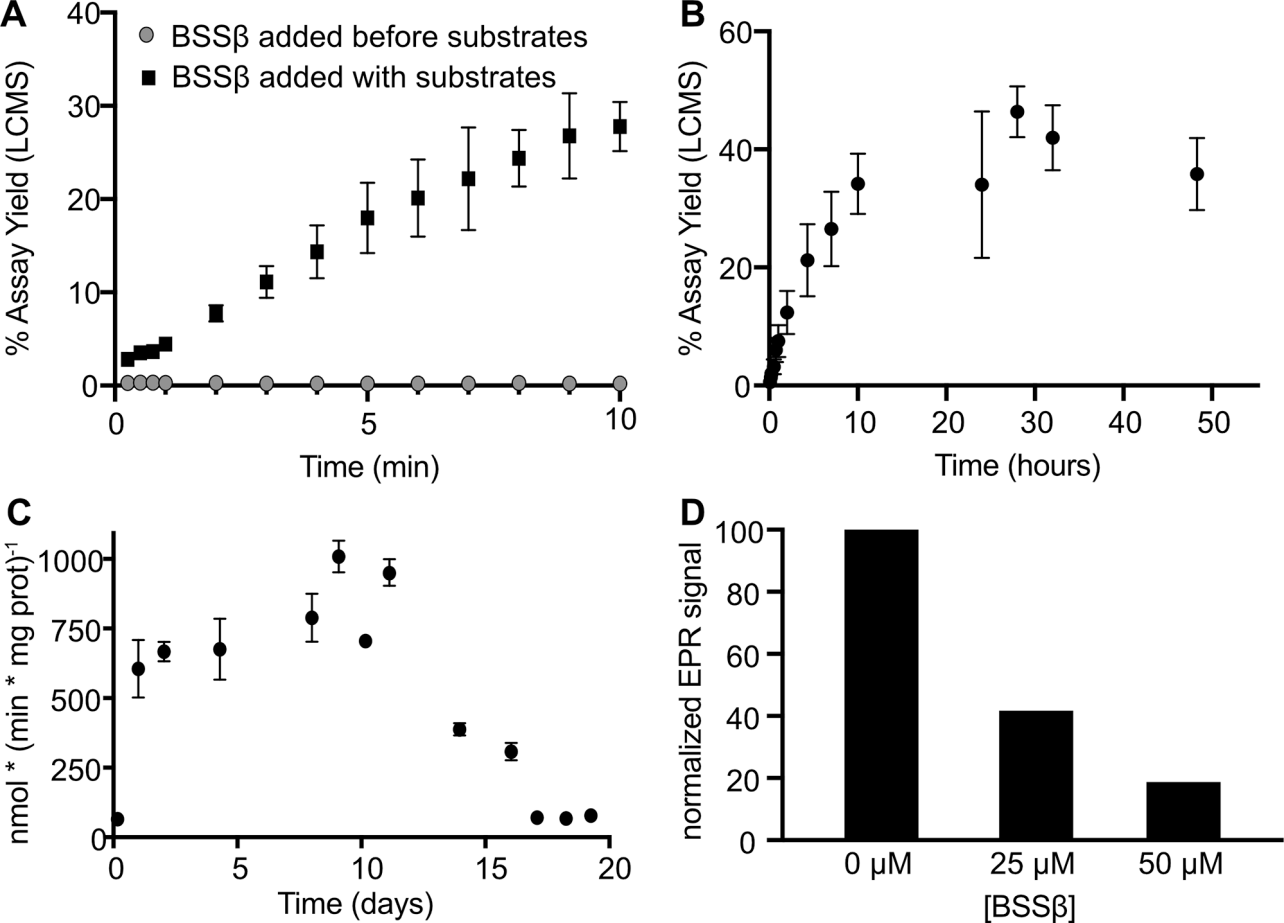
Reaction optimization leads to >340-fold increase in TTN. (A) Hydroalkylation time courses were conducted in triplicate with differing orders of addition. The same activation reaction was used for all time courses. In one condition, BSSαγ and BSSβ were briefly combined prior to substrate addition with the following order of addition: buffer, DTT (1 mM), BSSαγ (1 μM), BSSβ (80 μM), and substrates (2% toluene v/v, 2 mM fumarate). In the second condition, all components were added at the same concentrations, but BSSαγ was added last to initiate the reaction. (B) Catalyst lifetime under turnover conditions was extended to ∼10 h by optimizing both the order of BSSβ addition and its concentration. Time-course experiments were conducted in triplicate. BSSαγ (0.25 μM) was added to toluene (0.5%, v/v), fumarate (10 mM), DTT (1 mM), and BSSβ (10 μM) to initiate reactions. (C) The residual activity of an activation reaction was monitored over a 20-day period. Each data point represents the average of triplicate hydroalkylation rates (Figure S5). Time courses were initiated by adding BSSαγ (0.5 μM) to a reaction mixture containing toluene (2% v/v), fumarate (5 mM), DTT (1 mM), and BSSβ (40 μM). (D) An activation reaction was conducted for 24 h and subsequently divided into three samples. BSSβ was added to two of them at final concentrations of 25 and 50 μM. All samples were frozen 5 min after BSSβ addition. EPR spectra were collected, and double integrals were calculated using Xenon software.

### BSSαγ Retains Hydroalkylation Activity for 11 Days Following Activation

Because of the large effects BSSβ had on the catalyst lifetime, we investigated the stability of the glycyl radical prior to BSSβ addition and whether BSSβ influences radical concentration. To determine whether radical concentration changes prior to hydroalkylation, we activated BSSαγ in the absence of BSSβ and removed aliquots at various time points to perform hydroalkylation assays assessing residual activity (Figure 2C and Figure S5, Table S4). We observed similar hydroalkylation rates for 11 days, at which point a sharp decline in activity occurred. Notably, this loss of activity coincided with visible protein precipitation in the activation mixture, suggesting that protein instability rather than radical decay may be the limiting factor. Although these results are consistent with a long-lived glycyl radical, we cannot rule out the possibility of radical regeneration during the activation as AE is not removed at any point throughout these experiments. Nonetheless, our data indicate that the observed catalyst lifetime is not limited by radical decay prior to BSSβ or substrate addition.

To assess whether BSSβ affects radical stability, we activated BSSαγ and divided the reaction into three samples: one without BSSβ, one with 25 μM BSSβ, and one with 50 μM BSSβ. All reactions were frozen 5 min after BSSβ addition. The inclusion of BSSβ led to rapid loss of activity, with greater loss observed at the higher BSSβ concentration (Figure 2D, Table S5). These results suggest that BSSβ promotes catalyst inactivation, potentially through radical quenching.

### Efficient H/D Exchange Requires BSSβ

BSS currently represents the only case of a GRE that can be activated in the absence of an accessory subunit, namely, BSSβ. Many potential roles for BSSβ have been proposed, but because assays using purified enzymes were not feasible until recently, these roles have not been explored in depth. Given the dramatic effect of BSSβ on the catalyst lifetime, we sought to investigate its potential role in thiyl radical formation. We hypothesized that BSSβ might facilitate thiyl radical formation even in the absence of a substrate, which could lead to premature radical quenching. Structural analyses have suggested that BSSβ may shift the conformational equilibrium of BSSα toward a closed state, positioning the Gly and Cys residues in close proximity to facilitate hydrogen atom transfer (HAT).^15^ Recent computational studies propose that despite this spatial arrangement in static structures, HAT from Cys to Gly is unlikely to occur to any appreciable extent in the absence of substrates.^18^ This proposal that thiyl radical formation is triggered by substrate binding is mechanistically reasonable, as generating a reactive species in the absence of substrate could be detrimental to the enzyme if not immediately funneled into catalysis. We aimed to determine whether BSSβ directly contributes to thiyl radical formation and whether its role is dependent on the presence of substrate(s).

As the thiyl radical has never been directly observed for any GRE, we assessed its formation indirectly by using deuterium exchange studies. Upon activation, GREs exhibit a characteristic doublet EPR signal arising from the glycyl radical.^19^ When these activated enzymes are exchanged into the D_2_O buffer, this doublet collapses to a singlet, indicating incorporation of deuterium at the Cα position of Gly, forming [2-^2^H]-Gly·.^17,31^ Because the Cα–H bonds of Gly are not inherently exchangeable, this incorporation can occur only via HAT with the exchangeable S–H of the catalytic Cys residue. HAT between Gly and Cys is thought to be stereoselective, as suggested by slower deuterium incorporation relative to catalysis.^47^ However, the detection of the [2-^2^H]-Gly· species demonstrates that this process is not perfectly stereospecific. Previous EPR studies with BSS in D_2_O were conducted using enzyme produced by the native organism, with all subunits present.^20–22^ In those cases, the presence of the thiyl radical has been inferred from the collapse of the glycyl radical doublet to a singlet, consistent with H/D exchange at the Gly residue. With heterologously produced and purified BSS components and in vitro activation assays now in hand, we systematically evaluated which components are required for H/D exchange to occur, providing new mechanistic insight into thiyl radical formation in BSS.

In all deuterium exchange experiments, BSSαγ was activated under standard conditions in the absence of BSSβ and substrates. Following glycyl radical installation, the activation mixture was diluted 1:2 into buffer prepared with D_2_O, at which point BSSβ and/or fumarate were added to selected samples. To quantify deuterium incorporation at the α-position of glycine, EPR spectra were simulated using fixed Hamiltonian parameters (Table S6), varying only the relative contributions of [2-^1^H]-Gly· and [2-^2^H]-Gly· using linear least-squares. As a control, we allowed the [2-^1^H]-Gly·/[2-^2^H]-Gly· ratio to vary for spectra acquired in H_2_O; all converged to the expected 100% [2-^1^H]-Gly·.

We tested BSSαγ in the presence and absence of BSSβ and fumarate to evaluate their individual contributions to H/D exchange. Omitting BSSβ and substrates yielded minimal H/D exchange that could only be observed through simulations (Figure 3, Table S7). Crystallographic studies suggest that, without BSSβ, BSSαγ binds fumarate but not toluene.^34^ However, even in the presence of fumarate, only minimal exchange was observed (6% [2-^2^H]-Gly·). This slight exchange could be detected only through spectral simulation (Figure 3). These results suggest that thiyl radical formation does not occur in appreciable amounts in the absence of BSSβ. In contrast, when BSSβ was added to activated BSSαγ, significant exchange to [2-^2^H]-Gly· was observed, regardless of whether fumarate was present (Figure 3, Table S7).

**Figure 3:**
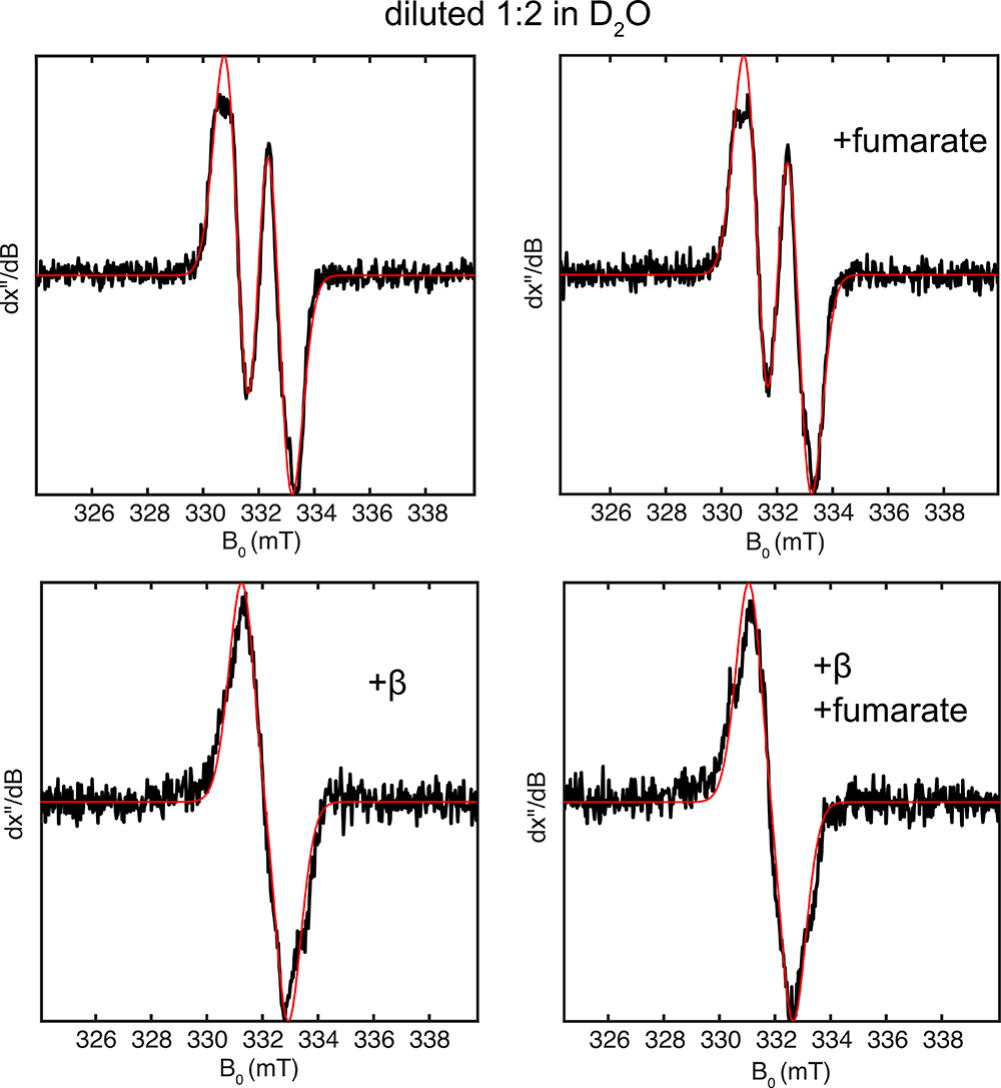
Normalized X-band EPR spectra (black) and simulations (red) from deuterium exchange studies of BSS. BSSαγ was activated in the absence of BSSβ and substrates, then divided into aliquots, and diluted 1:2 into buffer prepared with D_2_O. BSSβ and/or fumarate were added to select samples following dilution (17 μM and 10 mM final conc., respectively). After 5 min of incubation, samples were flash-frozen for EPR analysis. Simulations were performed by fixing the Hamiltonian parameters for [2^1^H]-Gly· and [2-^2^H]-Gly· (Table S6) and fitting their relative contributions using linear least-squares. Minimal deuterium incorporation was observed in BSSαγ with and without fumarate, and samples containing BSSβ showed significant exchange to [2-^2^H]-Gly·.

To test whether substrate binding influences thiyl radical formation, as has been proposed by computational studies,^18^ we reduced the extent of dilution in D_2_O (1:1 instead of 1:2) to slow deuterium incorporation and enhance our ability to detect differences between conditions. Using this slightly modified setup, we systematically varied the presence of BSSβ, fumarate, and toluene. When the spectra are simulated, we observed modest substrate-dependent effects without BSSβ. The addition of substrates slightly increased deuterium incorporation, increasing the percentage of [2-^2^H]-Gly· from 7 to 11–21% (Figure 4, Figure S6, Table S8). Consistent with results obtained under 1:2 dilution conditions, BSSβ had a much stronger effect: in the absence of substrates, its addition increased the percentage of [2-^2^H]-Gly· to 49%. Each substrate alone also enhanced incorporation (53 and 55% [2-^2^H]-Gly· with fumarate and toluene, respectively). When both substrates and BSSβ were present, deuterium incorporation was the highest, with 67% [2-^2^H]-Gly·.

**Figure 4:**
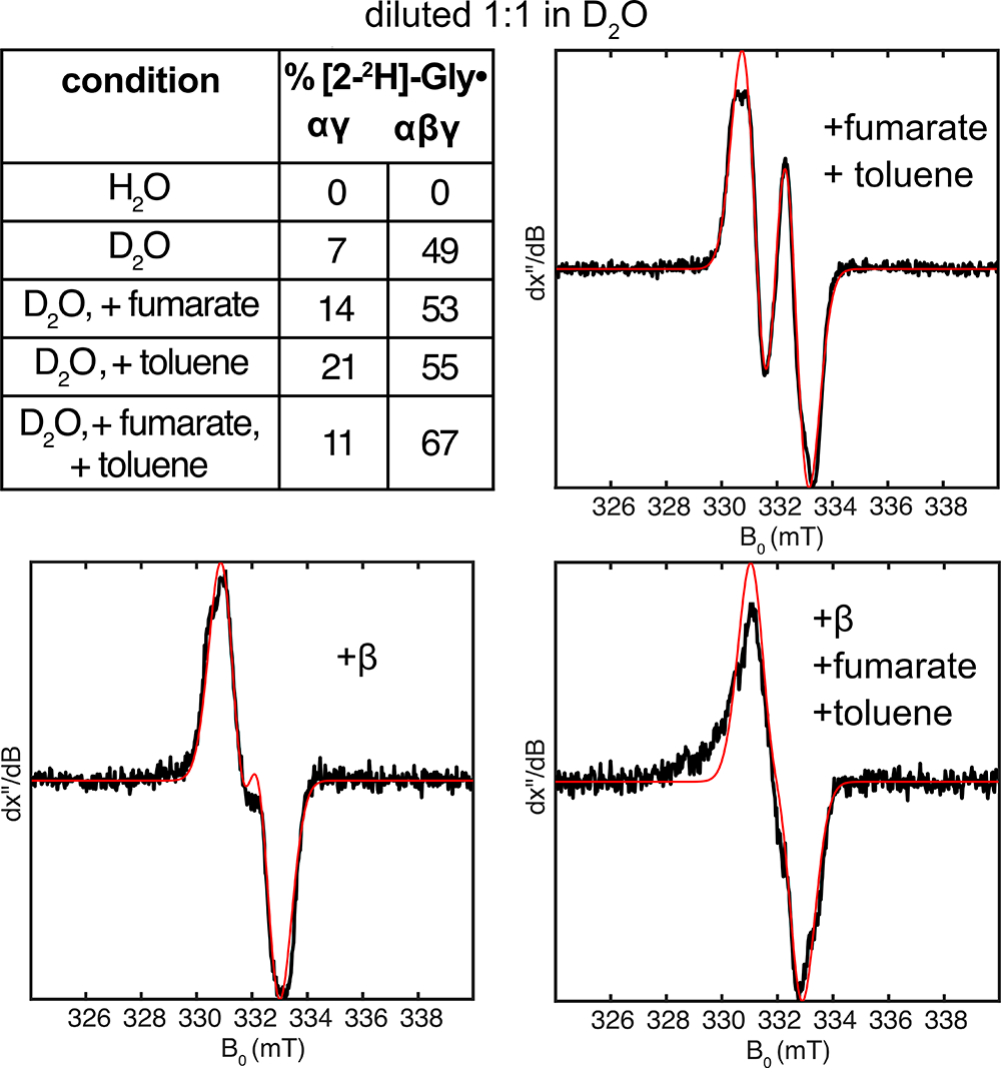
Normalized X-band EPR spectra (black) and simulations (red) from deuterium exchange studies of BSS at lower D_2_O dilution. BSSαγ was activated in the absence of BSSβ and substrates, then divided into aliquots, and diluted 1:1 into buffer prepared with D_2_O. BSSβ, fumarate, and/or toluene were added to select samples following dilution (25 μM, 10 mM, and 1% v/v final conc., respectively). After 5 min of incubation, samples were flash-frozen for EPR analysis. Simulations were performed by fixing the Hamiltonian parameters for [2-^1^H]-Gly· and [2-^2^H]-Gly· and fitting their relative contributions using linear least-squares. The percentage of [2-^2^H]-Gly· incorporation for each sample is shown in the table above. All spectra used to generate this table are shown in Figure S6.

Lastly, we sought to confirm that radical exchange with the active site Cys residue (Cys493) was responsible for the collapse of the doublet signal to a singlet, as has previously been shown for PFL.^47,48^ We created a C493A variant of BSSα and repeated EPR assays alongside wtBSSα as a control. BSSβ were included in these assays. As expected, no deuterium incorporation is observed in the C493A variant (Figure S7).

Taken together, these results show that BSSβ is necessary for significant thiyl radical formation and sufficient to promote it without substrate, contradicting prior computational modeling.^18^ However, substrate binding does appear to accelerate the process, consistent with that prediction. An alternative interpretation of these data could be that hydrogen atom transfer (HAT) is enantiospecific only in the absence of BSSβ, thereby preventing deuterium incorporation into glycine; however, this scenario seems less likely as BSSβ forms a more rigid, closed conformation. To our knowledge, the only other GRE that fails to produce a singlet upon incubation in D_2_O is anaerobic ribonucleotide reductase (aRNR).^49^ Moreover, the ability to regulate thiyl radical formation through an accessory subunit is, at present, unique to BSS among characterized GREs.

### Mutagenesis of BSSβ

To better understand how BSSβ regulates thiyl radical formation, we investigated structural features that may contribute to this function. The C-terminus of BSSβ is the only region that directly contacts the glycyl radical domain (GRD), suggesting a potential role in modulating conformational dynamics of the GRD and thus thiyl radical formation (Figure 1).^15^ To test this, we deleted the final seven residues of BSSβ and evaluated the impact on both the thiyl radical formation and overall hydroalkylation activity. In parallel, we generated a cluster-deficient BSSβ variant to examine the role of the Fe–S cluster.

To generate these BSSβ variants, we introduced a C29S mutation as previously described to create the cluster-deficient BSSβ-ΔFeS variant^33^ and an L75stop mutation to generate the C-terminal truncation variant BSSβ-ΔCterm based on structural analysis.^15,34^ Both mutants were expressed solubly and were readily purified; however, the BSSβ-ΔFeS variant appeared truncated via SDS-PAGE (Figure S8). When assessed using intact protein MS, no full length BSSβ-ΔFeS could be detected, further confirming truncation (Table S9). We attempted to obtain full-length BSSβ-ΔFeS by shortening expression times (3 h), adding protease inhibitors during purification, and performing purification at 4 °C, but none of the purified samples contained full-length BSSβ-ΔFeS. We next quantified Fe content within the variants using the ferene assay^40^ and found that BSSβ-ΔCterm retained cluster loading comparable to wild-type BSSβ (2.3 ± 0.4 and 3.0 ± 1.0 Fe/protein), while BSSβ-ΔFeS lacked appreciable Fe–S cluster incorporation (0.00 ± 0.01 Fe/protein), confirming disruption of cluster coordination as previously reported.^33^

Since BSSβ is highly prone to proteolysis without its [4Fe–4S] cluster, only the BSSβ-ΔCterm variant was used for further testing. The effects of wtBSSβ on thiyl radical formation were compared with those of BSSβ-ΔCterm using deuterium exchange. In comparison to wtBSSβ, thiyl radical formation was significantly diminished when the BSSβ-ΔCterm variant was added to activated BSSαγ (Figure 5, Figure S9, Table S10). Hydroalkylation assays mirrored these findings; reactions with BSSβ-ΔCterm formed only trace levels of product, as compared to the 100% assay yield observed for reactions with wtBSSβ (Figure 5, Table S11). These results are consistent with structural analyses suggesting that the C-terminus of BSSβ may regulate GRD conformational changes, thereby influencing thiyl radical formation.^15^

**Figure 5:**
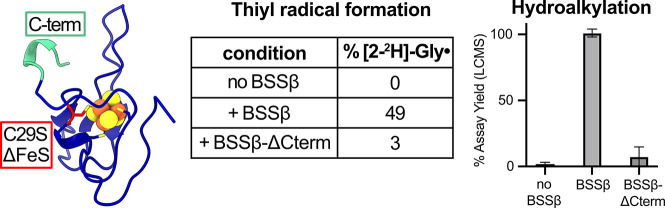
Mutagenesis of BSSβ to determine impact of C-terminus and [4Fe–4S] cluster. Two BSSβ variants were generated and purified, BSSβ-ΔCterm, which lacks the C-terminal residues (green), and BSSβ-ΔFeS, which contains a C29S mutation that disrupts [4Fe–4S] cluster binding (red). The BSSβ-ΔFeS is prone to proteolysis and could not be purified in its full-length form. Deuterium exchange assays were performed and were the BSSβ-ΔCterm variant as in Figure 4 in the absence of substrate. The same activation reaction was used for all of the hydroalkylations. Hydroalkylations were initiated by adding BSSαγ (1 μM) to a mixture containing toluene (1% v/v), fumarate (10 mM), DTT (1 mM), and either no BSSβ, wild-type BSSβ (10 μM), or BSSβ-ΔCterm (10 μM).

## Discussion

In many enzymatic systems, the formation of high-energy intermediates is gated or accelerated by substrate binding. For example, the Fe­(IV)-oxo species in cytochrome P450s and the 5′-deoxyadenosyl radical in radical SAM enzymes are typically accelerated upon substrate binding.^50–54^ However, both systems are known to undergo uncoupling under certain conditions, leading to off-pathway intermediate decay. In our reconstituted in vitro system, we observe a particularly prominent form of uncoupling in benzylsuccinate synthase (BSS), thiyl radical formation, that is triggered by the β subunit even in the absence of substrate, resulting in reduced radical lifetime. The extent of this uncoupling and the fact that it appears to be instigated by an accessory subunit is notable. It has been proposed that the [4Fe–4S] cluster in BSSβ could function as a hole acceptor (i.e., terminal reductant),^37,38^ and perhaps, if such a hole-hopping pathway exists, it may serve to quench unproductive thiyl radicals and return the enzyme to its inactivated state. Such a mechanism would be reminiscent of protective decay pathways proposed for P450s and other radical enzymes,^55^ although these have not been experimentally demonstrated in BSS and remain speculative. We also emphasize that the observations made in this study are made under in vitro conditions, where the relative concentrations and assembly states of α, β, and γ and the activating enzyme (AE) may not reflect those in the native cellular environment. It is possible that in vivo radical formation is more tightly regulated to prevent such uncoupling.

Regardless of the cellular context, understanding how BSSβ influences in vitro reactivity is critical for optimizing the catalytic performance. Although BSSγ appears to only affect catalyst solubility,^15,33^ we find that BSSβ has a strong impact not only on activity^10^ but also on reaction consistency and catalyst lifetime in vitro. As discussed above, we propose that this is due to BSSβ’s ability to trigger formation of the thiyl radical in the absence of substrate. In such cases, the thiyl radical could be quenched nonproductively, leading to catalyst inactivation. With this working hypothesis in mind, we revisit our previous interpretation of results that demonstrate lower glycyl radical incorporation in the presence of BSSβ.^10^ Instead of BSSβ inhibiting the formation of glycyl radical through stabilization of a closed state of BSSα, BSSβ could be affecting radical persistence. Distinguishing between these interpretations is not possible with the current data and will require future investigation. Nonetheless, by tuning the concentration of BSSβ and the order of its addition, we improved the total turnover number (TTN) more than 340-fold, from ∼50 to > 17,000. This enables a reduction in catalyst loading from 2 mol % to 0.01 mol % while maintaining >90% yields, a critical improvement for the practical implementation of these enzymes in engineering efforts and biocatalysis.

In addition to improving catalysis, we set out to clarify unresolved questions about BSSγ and BSSβ. As the cofactor of BSSγ had never been visualized, we determined a crystal structure of the full BSS complex with both BSSγ and BSSβ clusters intact. This structure revealed that the cluster and binding region of BSSγ closely resemble those of BSSβ, consistent with their shared HiPP-like fold (Figure 1). Beyond BSSβ and BSSγ, both HPAD and (1-methylalkyl)­succinate synthase (MASS) contain Fe–S cluster-bearing subunits.^36,56,57^ Many of these subunits share a conserved HiPP-like fold but may play distinct roles. We propose that the Fe–S clusters within GREs serve as a structural core, allowing the surrounding loops and termini to evolve independently to fulfill diverse functions across different GREs.

To the best of our knowledge, BSS is the only GRE in which thiyl radical formation is explicitly regulated by an accessory subunit. Why might this seemingly risky design have evolved? We propose that the β subunit is essential for forming a tightly sealed, hydrophobic active site that confers high affinity for toluene.^34,46^ This “toluene sponge” behavior enables BSS to scavenge low levels of toluene. Indeed, when using an anaerobic chamber without an activated carbon filter, we observe product formation in reactions even without toluene addition, implying that background toluene in our anaerobic chamber’s atmosphere is sufficient. This extraordinarily tight binding is likely critical in vivo, as the host organism can use toluene as its sole carbon source and relies on BSS to initiate its metabolism under anaerobic conditions.^4,6,35,58,59^ Other GREs act on more functionalized substrates that may help trigger radical formation upon binding. In contrast, BSS must overcome the hydrophobicity and volatility of toluene, and BSSβ likely evolved to meet this unique challenge.

## Conclusions

In summary, our studies reveal that BSSβ promotes thiyl radical formation even in the absence of a substrate, which in turn leads to significant radical quenching when a substrate is not present. This mechanistic insight allowed us to optimize the reaction conditions, boosting total turnover numbers more than 340-fold and extending catalyst longevity, thereby laying the groundwork for future biocatalytic applications of XSSs.

## Supplementary Material



## Notes

Additional materials, methods, experimental details, and experimental data, including EPR spectra, SDS-PAGE gel, and additional hydroalkylation assays (PDF)

## Notes

PDB ID: 9PZJ
