# Supplementary material for: Accessory Subunit Regulates Thiyl Radical Formation in Benzylsuccinate Synthase
Source: Biochemistry. 2025 Oct 13;64(21):4414–23. doi: 10.1021/acs.biochem.5c00492 (PMC12590467; doi:10.1021/acs.biochem.5c00492)
Supplement: Supplementary file 1 [file bi5c00492_si_001.pdf]

Supplementary Information

Accessory subunit regulates thiyl radical formation in benzylsuccinate synthase

Shukurah Anas,[1] Jian Liu,[1] Anshika Vats,[1] Rhea Gainadi,[1] Siraj Sharif,[1] Aiden Piriyatamwong,[1] Mary Catherine Andorfer[1,*]

[1]Department of Chemistry, Michigan State University, East Lansing, MI 48824, USA
* To whom correspondence may be addressed Mary C. Andorfer
Email: andorfe3@msu.edu (M.C.A.)

Supplemental methods:

Plasmid Construction and Mutagenesis

Details of plasmid construction for BSSαγ (UniProt ID: O68395 and O68394), BSSβ (UniProt ID: O68396) and IbsAE (UniProt ID: A0A096ZNX5) were published previously.[1–4] In previous studies, tutG (BSSβ) and tutH (chaperone protein) were cloned into pRSF-DUET, and an N-terminal His tag was added to both genes. This construct served as the template for generating the variants used in this study. The His tag was removed from tutH to enable efficient purification of wild-type BSSβ via IMAC. This construct was produced using In-Fusion Snap® Assembly Kit (Takara) using 5'-ACCATGGGCAGCAGCAGCCAGGATCCTAAAACCTAC-3' and 5'-GTAGGTTTTAGGATCCTGGCTGCTGCTGCCCATGGT-3' as the forward and reverse primers, respectively. The PCR product was purified over 0.7 % agarose gel agarose gel using an Omega Bio-Tek EZNA gel extraction kit. The purified PCR product was incubated with 5X In-fusion Snap® Assembly master mix (Takara) at 55 °C for 15 min, and the resulting mixture was transformed into Stellar competent cells (Takara).

BSSβ-ΔFeS variant: A variant of BSSβ that lacked the [4Fe–4S] cluster (BSSβ-ΔFeS) was previously described.[4] This variant contained a C29S mutation. The wild-type BSSβ construct described above was used as a template and the C29S mutation was introduced with the In-Fusion Snap® Assembly Kit (Takara) using 5'-ACGGCGAGGCCGTGCCGGAGTTCCAAATGG-3' and 5'-CATACCGAGGTGGGTACGGCGAGGCCGTGC-3' as the forward and reverse primers. The PCR product was purified over 0.7 % agarose gel agarose gel using an Omega Bio-Tek EZNA gel extraction kit. The purified PCR product was incubated with 5X In-fusion Snap® Assembly master mix (Takara) at 55 °C for 15 min. The resulting the reaction was transformed into Stellar competent cells (Takara).

BSSβ-ΔCterm variant: To truncate the C-terminus of BSSβ, a stop codon was introduced at position L75, yielding the BSSβ-ΔCterm variant lacking the final seven residues. The wild-type BSSβ construct was used as a template and the L75stop mutation was introduced with the In-Fusion Snap® Assembly Kit (Takara) using 5'-TTCCGCGACCACGTCTGACTCGAGTCTGGT-3' and 5'-GACGTGGTCGCGGAAACTTCATTTGCCTTC-3' as the forward and reverse primers, respectively. The PCR product was purified over 0.7 % agarose gel agarose gel using an Omega Bio-Tek EZNA gel extraction kit. The purified PCR product was incubated with 5X In-fusion Snap® Assembly master mix (Takara) at 55 °C for 15 min. The resulting the reaction was transformed into Stellar competent cells (Takara).

BSSα-C493A variant: The C493A mutation was introduced into BSSα using the BSSαγ plasmid described in Vats *et al.*[2] as a template, 5'-TGGGTCAACGTGCTGGCCATGTCGCCCGGC-3' and 5'-CAGCACGTTGACCCAGTTGTGGCTTCCTC-3' as the forward and reverse primers, respectively, and the In-Fusion Snap® Assembly Kit (Takara). The PCR product was purified over 0.7 % agarose gel agarose gel using an Omega Bio-Tek EZNA gel extraction kit. The purified PCR product was incubated with 5X In-fusion Snap® Assembly master mix (Takara) at 55 °C for 15 min. The resulting the reaction was transformed into Stellar competent cells (Takara). All constructs in this study were sequenced through whole-plasmid sequencing (plasmidsaurus.com).

Expression and purification

Expression and purification of proteins are described by Vats *et al*.[2] All constructs were transformed into T7 Express cells (New England Biolabs) and a single colony was used to make a glycerol stock of each. Starter cultures were inoculated from glycerol stocks and grown overnight at 37 °C at 220 rpm in LB containing antibiotics (100 µg/mL ampicillin for BSSαγ; 50 µg/mL kanamycin for IbsAE, wtBSSβ, and BSSβ variants). Expression cultures were inoculated with 10 mL of starter culture per 1 L of media (LB is used for BSSαγ and BSSβ; TB is used for

IbsAE). Expression media all contained the corresponding antibiotics. Expression media was also supplemented with iron (II) ammonium sulfate hexahydrate (150 mg/L, CAS: 7783-85-9) and L-cysteine (47 mg/L) for all constructs, except for the BSSβ-ΔFeS variant, which lacks the [4Fe–4S] cluster. Expression cultures were grown at 37°C at 220 rpm to an OD600 = 0.8–1, at which point they were cooled at 4 ˚C for 30 minutes and subsequently induced with IPTG (1 mM final conc., GoldBio). Induced cultures were expressed for 16–20 h at 22°C at 100 rpm. Cells were pelleted by centrifugation, flash frozen in liquid nitrogen, and stored at −80°C until lysis.

Cell lysis and protein purification were performed anaerobically in an MBraun chamber at 10–15 °C. Cell pellets were transferred into the anaerobic chamber while still frozen and thawed for 30–60 min prior to lysis. All buffers were sparged with nitrogen and transferred into the anaerobic chamber for use. For lysis of cells, cell paste from 2 L of culture was resuspended in 25 mL lysis buffer (50 mM HEPES pH 8.0, 300 mM NaCl, 10% glycerol, 1 mg/mL lysozyme (Sigma Aldrich), 25 µL of 2.5 MU/mL DNase I (from bovine pancreas, Sigma Aldrich)). Cells were resuspended by mashing cell paste with a spatula, after which cells were sonicated for 2 × 2 min cycles of 2 s on and 2 s off at an amplitude of 20 (Qsonica). Lysate was clarified by centrifugation at 14,000 × *g* for 45 min at 4 °C. All proteins were purified on Ni Sepharose Excel resin, which was gravity-packed into 10 mL plastic spin columns (Thermo Scientific™ Pierce™ Centrifuge Columns). Columns were equilibrated with 25 mL lysis buffer before passing cell lysate through by gravity before passing cell lysate through by gravity. Columns were washed with 25 mL of wash buffer (50 mM HEPES pH 8.0, 300 mM NaCl, 10 % glycerol, 10 mM imidazole) and eluted into new 50 mL falcon tubes with 2.5 mL of elution buffer (50 mM HEPES pH 8.0, 300 mM NaCl, 300 mM imidazole). Proteins were then buffer exchanged into activation/desalting buffer (50 mM HEPES pH 8.0, 300 mM NaCl), concentrated to ~200–500 µM, aliquoted and flash frozen. Iron quantification was conducted using a ferene assay[5] and iron standards (EINECS 231-714-2).

Hydroalkylation assays

Glycyl radical installations were conducted as described in the main text.[2] Hydroalkylations were conducted 3–24 hours after glycyl radical installation by adding DTT (1 mM final conc.), fumarate (2–10 mM final conc.), toluene (0.5–2% v/v), BSSβ (10–80 µM final conc.), and activated BSSαγ (0.25–2 µM final conc., added as activation reaction) diluted to the final reaction volume with activation buffer (50 mM HEPES pH 8.0, 300 mM NaCl). Specific reagent concentrations and order of addition are detailed in the corresponding figures and figure legends. All reactions were conducted in an MBraun anaerobic chamber in PCR or Eppendorf tubes with a final volume ranging from 25–500 µL for each reaction and incubated at 25 °C with no shaking. Hydroalkylation reactions were quenched with two volumes of methanol. Internal standard (2 mM 3-chlorobenzoic acid) was added in a 1:1 (v/v) ratio relative to the hydroalkylation reaction volume. Quenched reactions were removed from the MBraun and protein was pelleted by centrifugation (20 min at 3400 rpm). The resulting supernatant was diluted 1.5-fold with milli-Q water, filtered through a 0.22 µm filter, and used for LC-MS analysis. Samples were further diluted when necessary to ensure analyte concentrations fell within the linear detection range of the mass spectrometer.

LC-MS analysis for data in Figs. 2A, 2B, and S4 was conducted using a Waters Acquity TQ-D UPLC/MS/MS system equipped with reversed-phase Ascentis Express C18 2.1 × 50 mm column. Solvent A was milli-Q H_2O with 0.1% formic acid (LCMS grade, Honeywell Fluka, CAS 64-18-6), and solvent B was acetonitrile (HPLC grade, Sigma Aldrich, CAS 75-05-8). SIR traces were acquired for ions of m/z 207 and 155 in negative ion mode using the following method: 0-1 min, B = 1%; 1-3 min, B = 1-95%; 3-4 min, B = 95%; 4-4.01 min, B = 1%; 4.01-5 min, B = 1%. Flow rate was held constant at 0.4 mL/min.

LC-MS analysis for data in Figs. 2C, 5, S3, S5 was conducted using a Waters Acquity Premier UPLC–MS system equipped with reversed-phase CORTECS Premier C18+ 1.6 µm 2.1 × 50 mm column. Solvent A was milli-Q H_2O with 0.1% formic acid (LCMS grade, Fisher Chemical,

CAS 64-18-6), and solvent B was acetonitrile (HPLC grade, VWR Chemicals, CAS 75-05-8) with 0.1% formic acid. SIR traces were acquired for ions of m/z 207.10 and 155.10 in negative ion mode using the following method: 0-1.5 min, B = 5-95%; 1.5-2.3 min, B = 95%; 2.3-2.5 min, B = 95-5%; 2.5-3 min, B = 5%. Flow rate was held constant at 0.8 mL/min.

For quantification, the ratios of peak areas corresponding to the product ion (m/z 207.1) and the internal standard ion (m/z 155.1) were compared to the standard curve with known amounts of benzylsuccinate (Sigma Aldrich, CAS 884-33-3) and 3-chlorobenzoic acid (Sigma-Aldrich, CAS number: 535-80-8). Assay yields were defined as 100x[BS]/initial [fumarate].

Intact protein MS was carried out on a Waters Xevo G2-XS QTof interfaced with a Waters Acquity UPLC. Purified protein (5 uL) was injected onto a short online desalting column (1.0 x 10 mm, HyperSil Gold CN, Thermo). Protein was eluted using the following gradient: initial conditions were 98% A (0.1% formic acid in water) and 2% B (acetonitrile) and were held until 5 min with the first three minutes diverted to waste, ramp to 75% B at 10 min, hold at 75% B until 12 min, return to 98% A at 12.01 min and hold until 15 min. The column was held at 30 °C and the flow rate was 0.1 ml/min. Proteins were ionized by electrospray operating in positive ion mode with capillary voltage at 3 kV, cone voltage at 35 V, source temp 100 °C, desolvation temp at 350 °C, desolvation gas flow was 600 L/hr and cone gas flow was 25 L/hr. Mass spectra were acquired in continuum mode with a 1 second scan time across an m/z range of 200-2000. Protein mass spectra were deconvoluted to give a neutral mass of the intact proteins using the MaxEnt I algorithm in the Masslynx software. Deconvoluted masses are shown in Table S8.

Supplemental Figures and Tables:

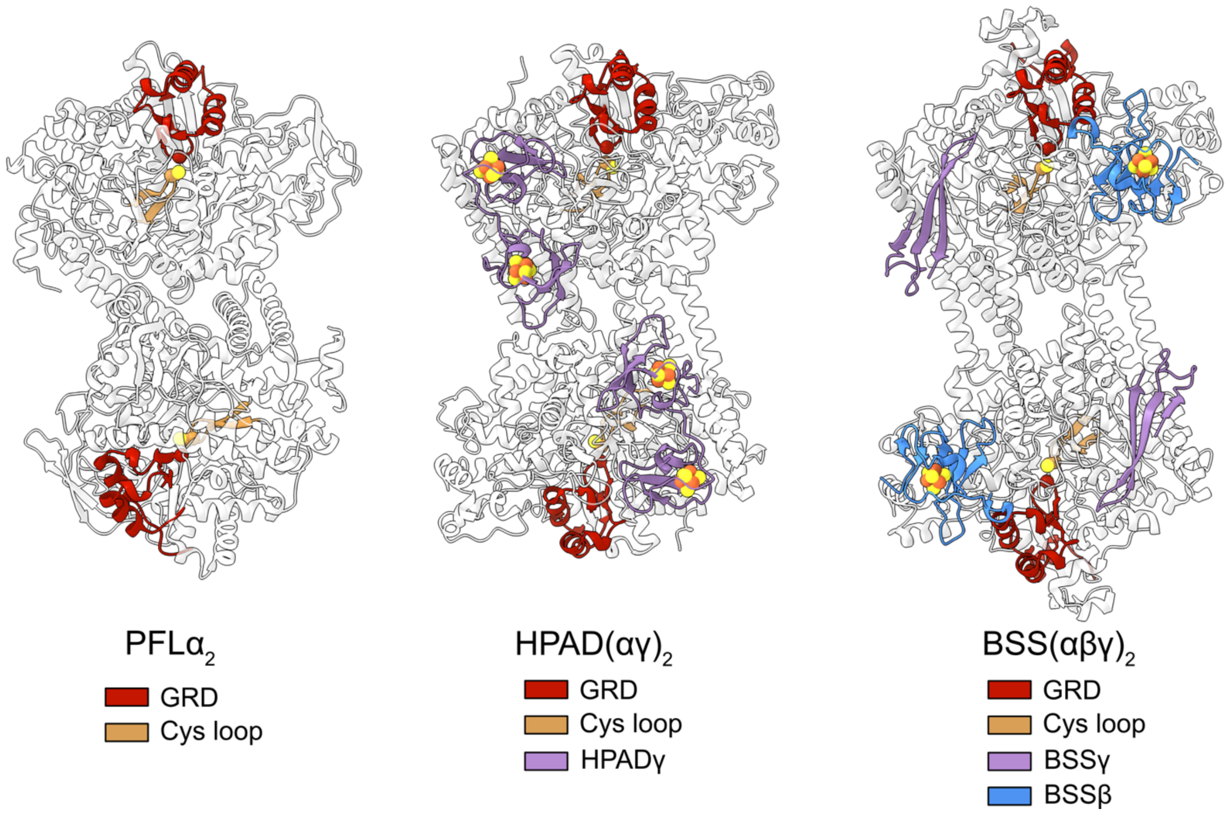

Figure S1*:* Subunit comparison of three GREs. All GREs contain a flexible glycyl radical domain (GRD, red) that harbors the essential glycine residue (red sphere). When the GRD is in a closed conformation, as is shown in these structures, the essential Gly residue lies in close proximity to the Cys loop (orange), which contains the catalytic Cys residue (shown as spheres). The active site of the GRE is near this Cys residue within the 10-stranded β/α-barrel. PFL, like most GREs, consists of a single catalytic subunit. HPAD consists of a complex between the catalytic α subunit and an accessory subunit (γ, purple) that binds two [4Fe–4S] clusters. BSS consists of a complex between the catalytic α subunit and two accessory subunits (γ, purple and β, blue), each of which bind an [4Fe–4S] cluster. PFL PDB ID: 1H18;[6] HPAD PDB ID: 2Y8N;[7] BSS PDB ID: 5BWE.[8]

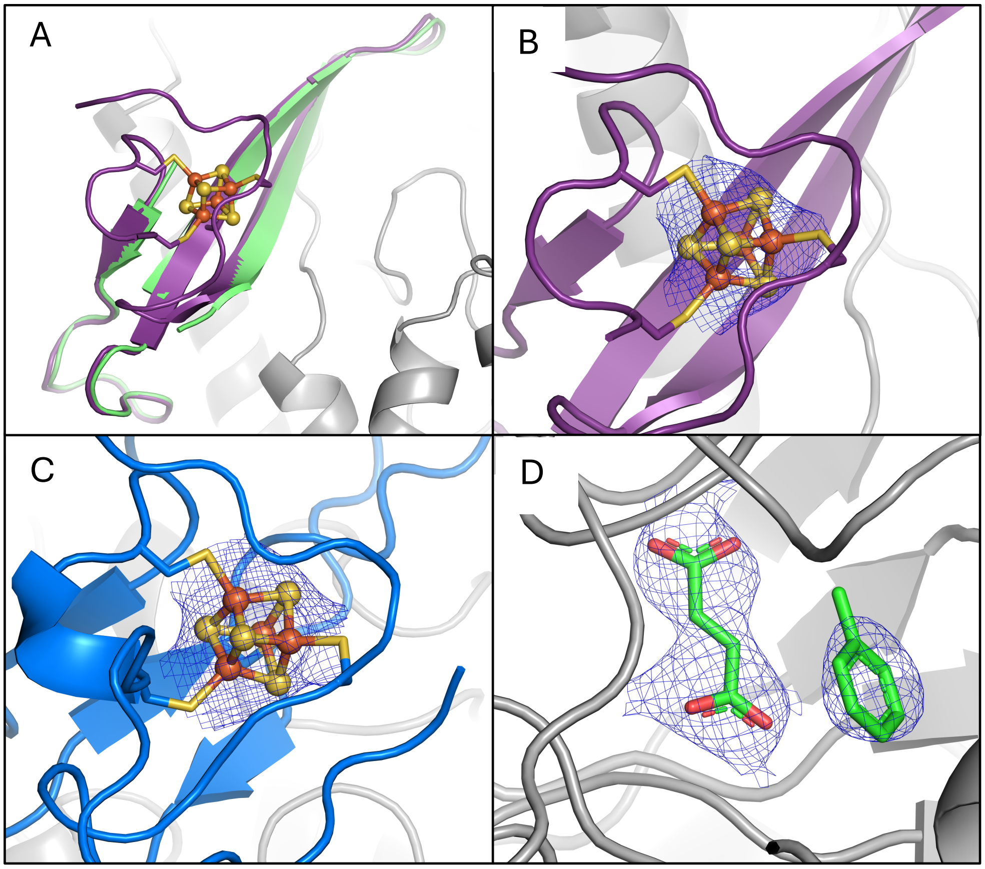

Figure S2: BSSγ and BSSβ each bind a [4Fe–4S] cluster, while BSSα binds fumarate and toluene as substrates in the active site. A) BSSγ from the structure solved herein (purple) overlaid with a previous structure of BSSγ that is lacking the N-terminus, C-terminus, and [4Fe–4S] cluster (green).[8] B) The 2mFo-DFc composite omit map contoured to 1σ is shown for the [4Fe–4S] cluster of BSSγ. C) The 2mFo-DFc composite omit map contoured to 1σ is shown for the [4Fe–4S] cluster of BSSβ. D) The 2mFo-DFc composite omit map contoured to 1σ is shown for fumarate and toluene, bound in the active site of BSSα.

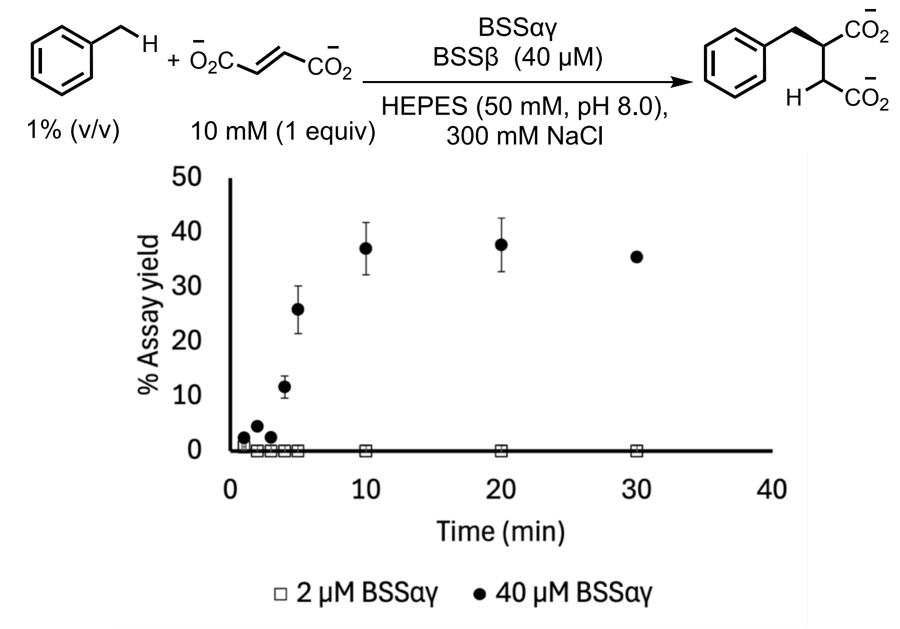

Figure S3: Time courses comparing catalyst lifetimes of BSS in hydroalkylations when BSSβ is added before substrates. Activated BSSαγ was added to buffer (final conc.: 2 μM [empty squares] and 40 μM [filled circles]), followed by BSSβ. The resulting solution was gently mixed, followed by the addition of fumarate and toluene. Assays were performed in triplicate.

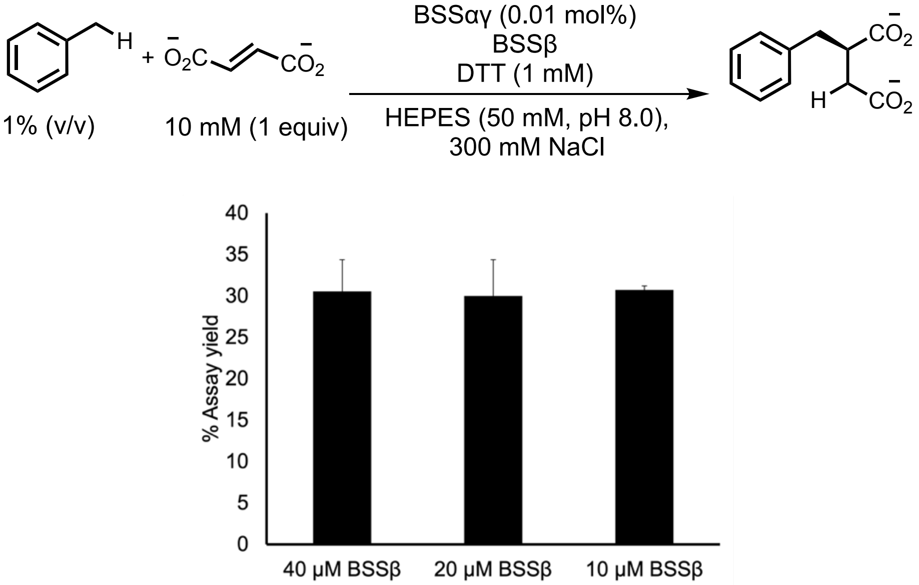

Figure S4: **Comparison of hydroalkylations with varying concentrations of BSSβ**. Fumarate and toluene were added to buffer and DTT. BSSβ was then added at different concentrations (10–40 μM), followed by activated BSSαγ (1 μM) to initiate catalysis. Reactions were carried out with a total volume of 100 μL and quenched after 1 hour. Assays were performed in triplicate.

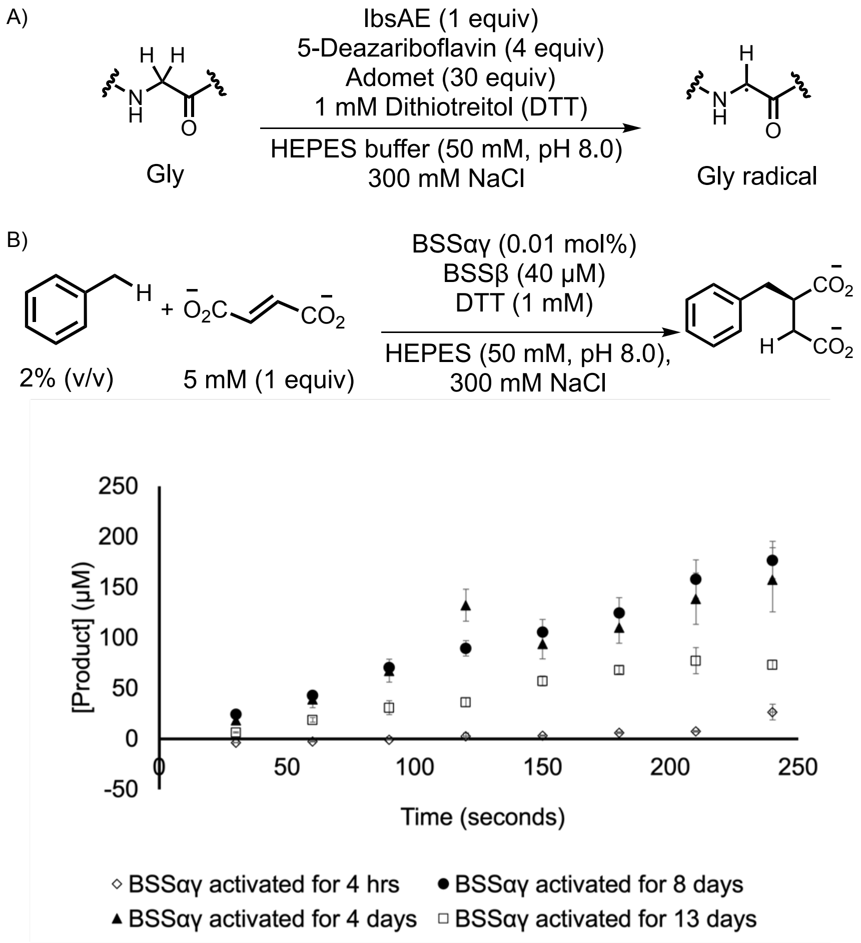

Figure S5: Representative time courses used to determine hydroalkylation activities in Fig. 2C. (A) BSSαγ was activated as described in the glycyl radical installation procedure in Methods Section "Activations" of the main text. (B) At specific time points after activation had been initiated, aliquots of the activated BSSαγ were used to assess the activity of BSSαγ. In all assays, fumarate and toluene were added to a mixture of buffer and DTT. BSSβ was then added, followed by activated BSSαγ (0.5 μM) to initiate catalysis. Assays were performed in triplicate.

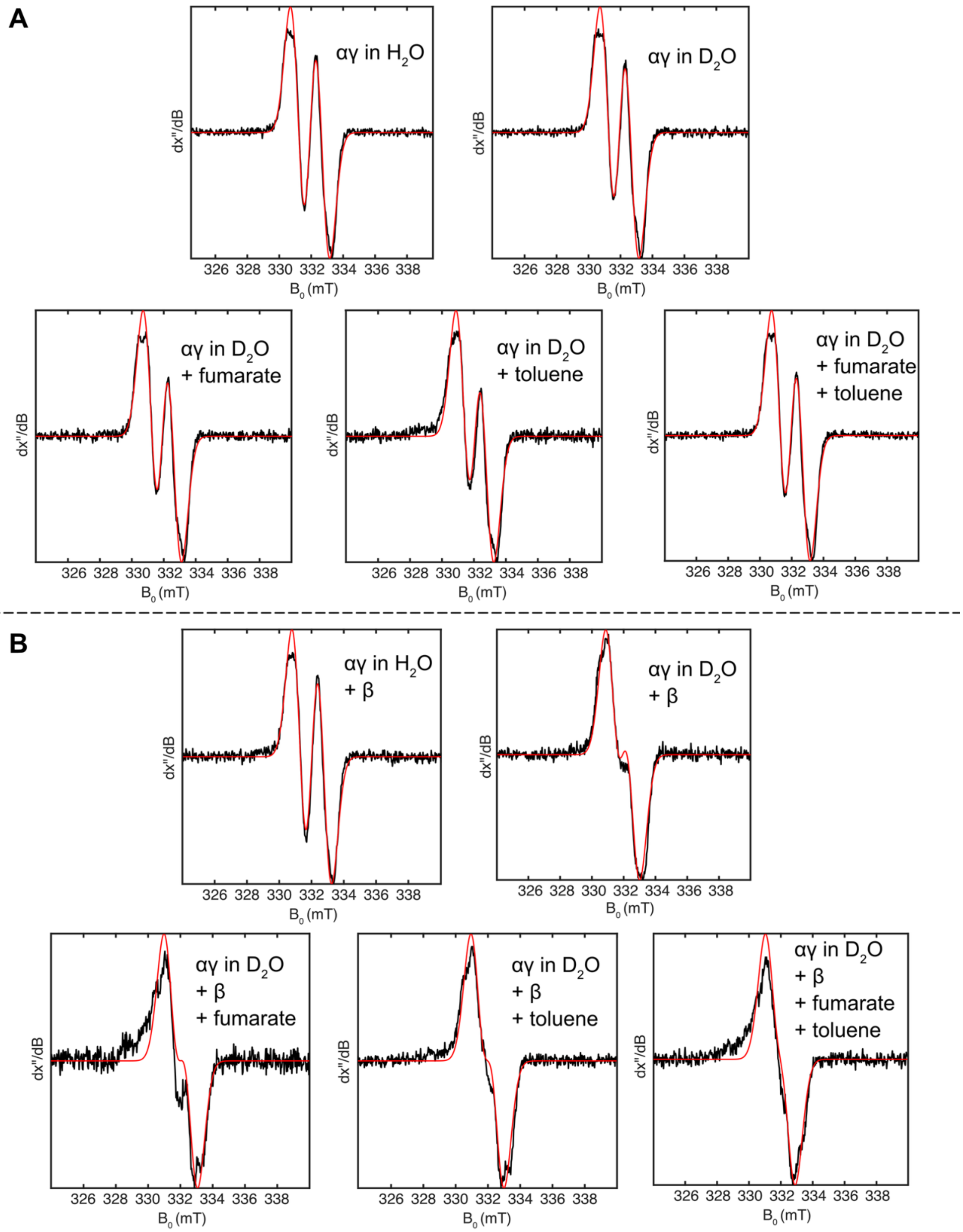

Figure S6: Normalized X-band EPR spectra (black) and simulations (red) from deuterium exchange studies of BSS. BSSαγ was activated in the absence of BSSβ and substrates, then divided into aliquots and diluted 1:1 into buffer prepared with D_2O. BSSβ, fumarate, and/or toluene were added to select samples following dilution. After 5 minutes of incubation, samples were flash-frozen for EPR analysis. Simulations were performed by fixing the Hamiltonian parameters for [2-^{1}H]-Gly• and [2-^{2}H]-Gly• and fitting their relative contributions using linear least-squares in EasySpin. The percentage of [2-^{2}H]-Gly• incorporation for each sample is shown in Fig. 4 of the main text, as well as Table S8.

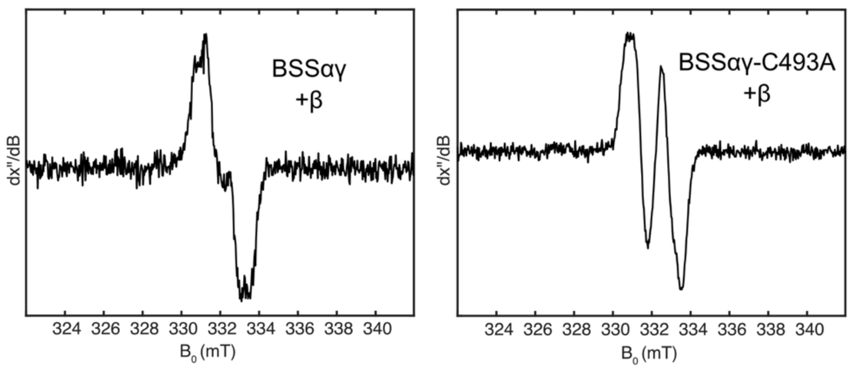

Figure S7: X-band EPR spectra of wtBSSα and BSSα variant C493A. wtBSSαγ (left) and BSSαγ-C493A (right) were activated in the absence of BSSβ, then diluted 1:1 into buffer prepared with D_2O containing 5 mM DTT. BSSβ was added following dilution. After 10 minutes of incubation, samples were flash-frozen for EPR analysis.

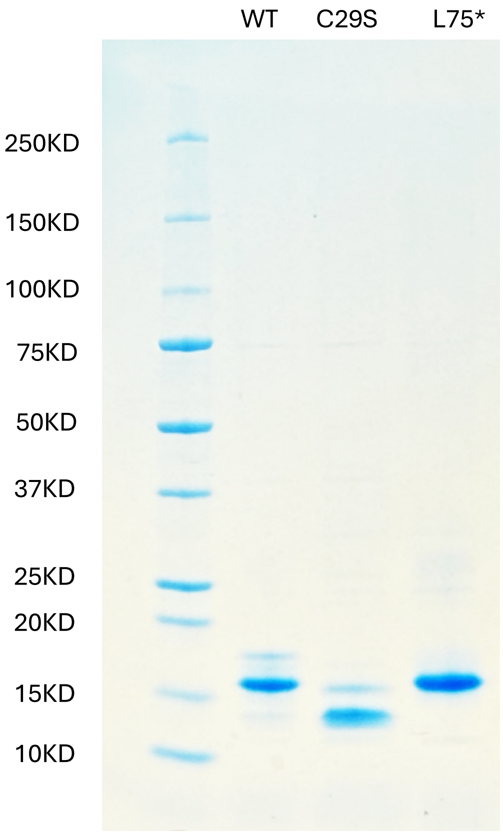

Figure S8: Gel of purified BSSβ variants.

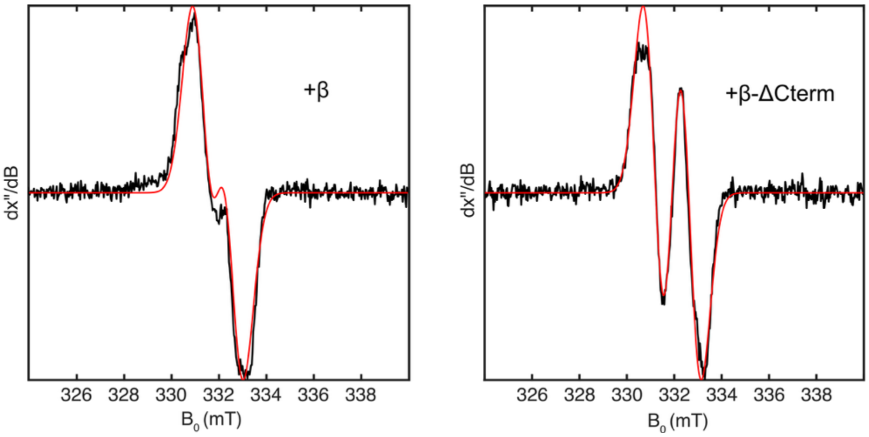

Figure S9: EPR Normalized X-band EPR spectra (black) and simulations (red) from deuterium exchange studies using wtBSSβ and BSSβ-ΔCterm. BSSαγ was activated in the absence of BSSβ and substrates, then divided into aliquots and diluted 1:1 into buffer prepared with D_2O. BSSβ was added to samples following dilution. After 5 minutes of incubation, samples were flash-frozen for EPR analysis. Simulations were performed by fixing the Hamiltonian parameters for [2-^{1}H]-Gly• and [2-^{2}H]-Gly• and fitting their relative contributions using linear least-squares in EasySpin. The percentage of [2-^{2}H]-Gly• incorporation for each sample is shown in Fig. 5 of the main text, as well as Table S9.

Table S1: Data collection and refinement statistics for BSSαβγ.

**Data collection and processing**	
Beamline	NSLS II NYX (19-ID)
Space group	$P2_12_12$
Unit cell dimensions	a=146.4 Å, b=118.5 Å, c=124.9 Å<br>a= 90.0°, b=90.0°, g=90.0°
Wavelength (Å)	0.979338
Resolution (Å)	33.68-2.9
Unique reflections*	48786 (4793)
Redundancy	13.7 (14.5)
Completeness (%)	100 (100)
<I>/<σI>	9.6 (2.3)
R_{merge} (%)	7.7 (33.0)
$CC_{1/2}$	99.2 (73.5)
**Model refinement**	
Resolution (Å)	33.68-2.9
R_{work} (%)	21.3
R_{free}** (%)	24.6
Model composition	
Non-hydrogen atoms	15,905
Residues	1,961
4Fe–4S clusters (SF4)	4
Fumarate (FUM)	2
Toluene (MBN)	2
Water molecules	13
Rmsd	
Bond length (Å)	0.003
Bond angle (°)	0.569
Average B factors ($Å^2$)	45.2
BSSα	43.2
BSSβ	54.3
BSSγ	64.1
Water	34.5
Rotamer outliers (%)	2
Ramachandran plot (%)	97.64%
Most favored	97%
Additionally allowed	3%
Disallowed	0%

*Highest resolution shell values are shown in parentheses.

**5% of reflections were selected as cross examination set.

Table S2: Table of values plotted in Fig. 2A.

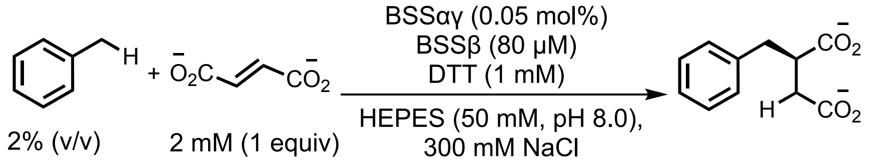

Time (minutes)	BSSβ added before substrates		BSSβ added with substrates	
	Average % assay yield	Standard deviation	Average% assay yield	Standard deviation
0.25	0.3	0.1	2.8	0.5
0.5	0.3	0.1	3.5	0.6
0.75	0.3	0.1	3.6	0.3
1	0.3	0.1	4.4	0.3
2	0.3	0.1	7.8	0.9
3	0.2	0.1	11.1	1.7
4	0.2	0.0	14.3	2.8
5	0.2	0.0	18.0	3.8
6	0.2	0.1	20.1	4.1
7	0.2	0.0	22.2	5.5
8	0.3	0.1	24.4	3.0
9	0.2	0.1	26.8	4.6
10	0.2	0.0	27.8	2.6

Table S3: Table of values plotted in Fig. 2B.

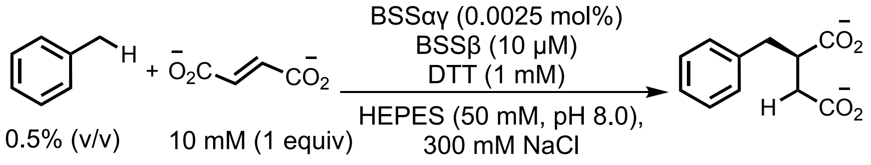

Time (hours)	Average % assay yield	Standard deviation
0.1	0.6	0.0
0.2	1.4	0.3
0.3	2.0	0.6
0.5	3.2	1.3
0.8	6.0	2.1
1.0	7.5	2.7
2.0	12.4	3.7
4.3	21.2	6.1
7.0	26.5	6.3

10.0	34.2	5.1
24.0	34.0	12.4
28.0	46.4	4.3
32.0	42.0	5.5
48.3	35.8	6.1

Table S4: Table of values plotted in Fig. 2C.

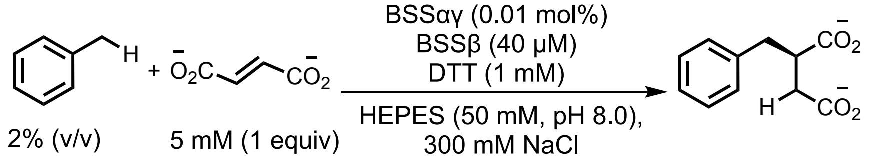

Days	Activity (nmol* (min*mg prot)$^{-1}$)	Standard deviation
0	71.3	5.9
1	605.3	103.0
2	666.8	34.9
4	675.6	109.6
8	788.7	86.5
9	1008.9	57.3
10	711.0	9.3
11	951.0	47.9
14	387.8	22.1
16	307.6	31.4
17	76.7	7.0
18	73.8	11.6
19	83.7	6.6

Table S5: Table of values plotted in Fig. 2D.

[BSSβ] (μM)	Double integral of EPR spectra	Normalized EPR signal
0	5.4	100.0
25	2.2	40.6
50	1.0	18.4

Table S6: EPR simulation parameters for [2-^{1}H]-Gly• and [2-^{2}H]-Gly•.

Species	g_{iso}	A (MHz)	lwpp (mT)
[2-^{1}H]-Gly•	2.00360 (2)	40.9 (2)	1.01 (1)
[2-^{2}H]-Gly•	2.00360	12.6 (5)	0.89 (3)

Errors, reported in parentheses on the last significant digit, represent the estimated 1σ uncertainties from the EasySpin non-linear least-squares fits. g_{iso} was fixed for the [2-^{2}H]-Gly• simulation.

Table S7: Estimated weights of [2-^{1}H]-Gly• and [2-^{2}H]-Gly• through simulations of EPR spectra in Figure 3.

Conditions	Fraction of [2-^{1}H]-Gly•	Fraction of [2-^{2}H]-Gly•
BSSαγ	0.96 (1)	0.036 (8)
BSSαγ +fumarate	0.94 (1)	0.062 (7)
BSSαγ + BSSβ	0.24 (1)	0.76 (1)
BSSαγ + BSSβ + fumarate	0.19 (1)	0.81 (1)

Simulations were performed by fixing the Hamiltonian parameters for [2-^{1}H]-Gly• and [2-^{2}H]-Gly• (Table S6) and fitting their relative contributions using linear least-squares in EasySpin. Errors, reported in parentheses on the last significant digit, represent the estimated 1σ uncertainties from the residual-based analysis.

Table S8: Estimated weights of [2-^{1}H]-Gly• and [2-^{2}H]-Gly• through simulations of EPR spectra in Figure 4 and S6.

Conditions	Fraction of [2-^{1}H]-Gly•	Fraction of [2-^{2}H]-Gly•
BSSαγ (H_2O)	1.00 (0)	0.00
BSSαγ	0.93 (1)	0.066 (6)
BSSαγ + fumarate	0.86 (1)	0.14 (1)
BSSαγ + toluene	0.79 (1)	0.21 (1)
BSSαγ + fumarate + toluene	0.89 (0)	0.11 (0)
BSSαγ + BSSβ (H_2O)	1.0 (0)	0.00
BSSαγ + BSSβ	0.51 (0)	0.49 (0)
BSSαγ + BSSβ + fumarate	0.47 (1)	0.53 (1)
BSSαγ + BSSβ + toluene	0.45 (1)	0.55 (1)
BSSαγ + BSSβ + fumarate + toluene	0.33 (1)	0.67 (1)

Simulations were performed by fixing the Hamiltonian parameters for [2-^{1}H]-Gly• and [2-^{2}H]-Gly• (Table S6) and fitting their relative contributions using linear least-squares in EasySpin. Errors, reported in parentheses on the last significant digit, represent the estimated 1σ uncertainties from the residual-based analysis.

Table S9: Deconvoluted intact masses for wtBSSβ and BSSβ variants.

Purified protein	Expected Mass (Da)	Major peak(s)
wtBSSβ	11,578	11,578
BSSβ-ΔCterm	10,723	10,722
BSSβ-ΔFeS	11,561	10,597<br>9,759<br>7,370

Table S10: Estimated weights of [2-^{1}H]-Gly• and [2-^{2}H]-Gly• through simulations of EPR spectra in Figure 5 and S8.

Conditions	Fraction of [2-^{1}H]-Gly•	Fraction of [2-^{2}H]-Gly•
BSSαγ	1.00 (0)	0.0014 (42)
BSSαγ + BSSβ	0.51 (0)	0.49 (0)
BSSαγ + BSSβ-ΔCterm (C29S)	0.97 (1)	0.025 (7)

Simulations were performed by fixing the Hamiltonian parameters for [2-^{1}H]-Gly• and [2-^{2}H]-Gly• (Table S6) and fitting their relative contributions using linear least-squares in EasySpin. Errors, reported in parentheses on the last significant digit, represent the estimated 1σ uncertainties from the residual-based analysis.

Table S11: Table of values for hydroalkylations plotted in Fig. 5.

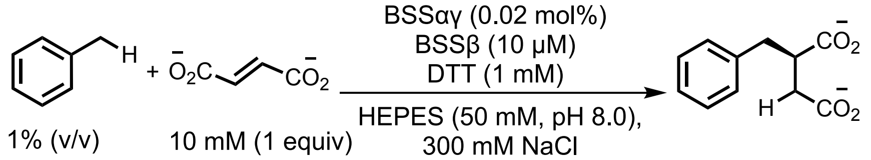

Variant	Average % assay yield	Standard deviation
No BSSβ	1.9	1.3
wtBSSβ	101.0	3.0
BSSβ-ΔCterm	7.4	7.4

Supplemental References

(1) Andorfer, M. C.; King-Roberts, D. T.; Imrich, C. N.; Brotheridge, B. G.; Drennan, C. L. Development of an in Vitro Method for Activation of X-Succinate Synthases for Fumarate Hydroalkylation. *iScience* **2023**, *26* (6), 106902. https://doi.org/10.1016/j.isci.2023.106902.

(2) Vats, A.; Anas, S.; Liu, J.; Chakraborty, A.; Ryu, J.; Mann, S.; Andorfer, M. C. Activation of X-Succinate Synthases for Fumarate Hydroalkylation Using an in Vitro Activation Method. *Bio-Protoc.* **2025**.

(3) Funk, M. A.; Judd, E. T.; Marsh, E. N. G.; Elliott, S. J.; Drennan, C. L. Structures of Benzylsuccinate Synthase Elucidate Roles of Accessory Subunits in Glycyl Radical Enzyme Activation and Activity. *Proc. Natl. Acad. Sci.* **2014**, *111* (28), 10161–10166. https://doi.org/10.1073/pnas.1405983111.

(4) Li, L.; Patterson, D. P.; Fox, C. C.; Lin, B.; Coschigano, P. W.; Marsh, E. N. G. Subunit Structure of Benzylsuccinate Synthase. *Biochemistry* **2009**, *48* (6), 1284–1292. https://doi.org/10.1021/bi801766g.

(5) Hennessy, D. J.; Reid, G. R.; Smith, F. E.; Thompson, S. L. Ferene — a New Spectrophotometric Reagent for Iron. *Can. J. Chem.* **1984**, *62* (4), 721–724. https://doi.org/10.1139/v84-121.

(6) Becker, A.; Kabsch, W. X-Ray Structure of Pyruvate Formate-Lyase in Complex with Pyruvate and CoA. *J. Biol. Chem.* **2002**, *277* (42), 40036–40042. https://doi.org/10.1074/jbc.m205821200.

(7) Martins, B. M.; Blaser, M.; Feliks, M.; Ullmann, G. M.; Buckel, W.; Selmer, T. Structural Basis for a Kolbe-Type Decarboxylation Catalyzed by a Glycyl Radical Enzyme. *J. Am. Chem. Soc.* **2011**, *133* (37), 14666–14674. https://doi.org/10.1021/ja203344x.

(8) Funk, M. A.; Marsh, E. N. G.; Drennan, C. L. Substrate-Bound Structures of Benzylsuccinate Synthase Reveal How Toluene Is Activated in Anaerobic Hydrocarbon Degradation. *J. Biol. Chem.* **2015**, *290* (37), 22398–22408. https://doi.org/10.1074/jbc.M115.670737.
